# Emergent 2D van der Waals materials photonic sources

**DOI:** 10.1515/nanoph-2024-0702

**Published:** 2025-03-11

**Authors:** Kwok Kwan Tang, Chun Li, Changhai Zhu, Peipei Li, Liyun Zhao, Qing Zhang

**Affiliations:** School of Materials Science and Engineering, 12465 Peking University, Beijing 100871, China; School of Physics and Electronic Engineering, Chongqing Normal University, Chongqing 401331, China; International School for Optoelectronic Engineering, Qilu University of Technology (Shandong Academy of Sciences), Jinan 250353, China

**Keywords:** 2D van der Waals semiconductors, light sources, exciton, exciton-polariton, nonlinear optics

## Abstract

Over the past two decades, two-dimensional (2D) van der Waals (vdW) semiconductors have garnered significant attention in the field of light sources due to their unique optoelectronic properties, such as high excitonic binding energy, tunable bandgaps, and strong optical anisotropy. These properties make 2D vdW semiconductors highly promising for next-generation light sources, offering advantages like enhanced efficiency, wavelength tunability, and polarization control. In this review, we summarize the development of various 2D vdW material-based light sources and their modulation mechanisms. We first provide an overview of excitonic properties and light-emission principles that aim to develop light sources with low-power, high-efficiency. Next, we discuss advances in 2D semiconductor lasers, including intralayer and interlayer exciton lasers, cavity-free systems, and exciton-polariton sources. We then look into single-photon emission and their integration into on-chip systems, followed by studies on nonlinear optical properties like high-order harmonic generation and P-band emission. Additionally, we cover advancements in electrically pumped light sources. The review concludes with an outlook on future developments of 2D vdW semiconductor light sources.

## 1 Introduction

Currently, the light source field is advancing toward high-efficiency and low-power consumption, tunable light sources, miniaturization and integration, application of new materials, flexible and wearable devices, nanophotonics and plasmonics, and quantum light sources – all of which are crucial for modern communication, display, and energy applications. Two-dimensional (2D) materials offer unique advantages in developing light sources due to their direct bandgap properties [1], high excitonic binding energy [2], tunable bandgaps [3], strong optical anisotropy [4], high mechanical flexibility and transparency [5], ability to form van der Waals (vdW) heterostructures [6], and compatibility with existing manufacturing processes [7]. These characteristics make 2D vdW materials highly promising for next-generation high-performance, low-power, and multifunctional light sources. This field, pioneered by the discovery of graphene [8], has since expanded rapidly to other promising 2D vdW materials like transition-metal dichalcogenides (TMDs), black phosphorus (BP), and hexagonal boron nitride (hBN), each offering distinct advantages for optoelectronic applications [9]. Monolayer TMDs, for example, including MoS_2_ and WS_2_, exhibit direct bandgaps [1], [3], [10] – unlike their bulk counterparts, which are indirect – resulting in enhanced photoluminescence (PL) and thus making them ideal for light emitting diodes (LEDs) [11] and nanolasers [12]. The atomically thin structure and unique optical properties of 2D vdW materials position them as prime candidates for low-power, high-efficiency electronic and optoelectronic devices, particularly where miniaturization is crucial [13], [14], [15], [16]. Furthermore, a particularly exciting application of 2D semiconductors is in single-photon sources, which are integral to quantum information science. Single-photon sources technology utilizing 2D vdW materials, such as TMDs and hBN, allows for on-demand single-photon generation with high purity and efficiency [17], [18], [19], [20]. For instance, hBN defects are capable of producing stable, room-temperature single-photon emissions (SPEs), making them promising for quantum optoelectronics [17] and quantum sensor [21]. With the growing need for compact, scalable, and high-performance quantum photonic systems, single-photon sources based on 2D vdW materials offers an exceptional pathway for integrating quantum capabilities with traditional photonic devices. Additionally, 2D semiconductors exhibit nonlinear optical phenomena such as high-order harmonic generation (HHG) and P-band emission, which hold significant promise for the development of tunable, integrable light sources, on-chip photonic circuits, ultrafast lasers, and advanced signal processing technologies [22], [23], [24], [25], [26]. For example, the γ-phase structure of InSe, characterized by its out-of-plane dipole orientation, enhances the efficiency of exciton scattering, enabling P-band emission at low excitation densities [27]. This nonlinear optical behavior in 2D vdW materials opens new avenues for photonic technologies that are both adaptable and compact, addressing critical needs in modern electronics and photonics.

In this review, we systematically summarize various types of light sources based on 2D layered vdW materials and their modulation mechanisms. First, we will provide a brief overview of the exciton properties and light-emission principles of vdW materials. Second, we will discuss the development of 2D semiconductor lasers, including recent advancements in intralayer and interlayer exciton lasers, lasers that operate without external cavities, and exciton-polariton (EP) emission sources. Third, we introduce research on SPE sources based on 2D vdW materials, with an emphasis on their integration into on-chip systems. Fourth, we will describe studies on the nonlinear optical properties of vdW materials, covering HHG and modulation, as well as P-band emission. Fifth, we will discuss the research and advancements in electrically pumped light sources based on vdW materials. Finally, we will offer an outlook on the future development of vdW materials light sources.

## 2 Emission properties of 2D vdW semiconductors

**Figure 1:**
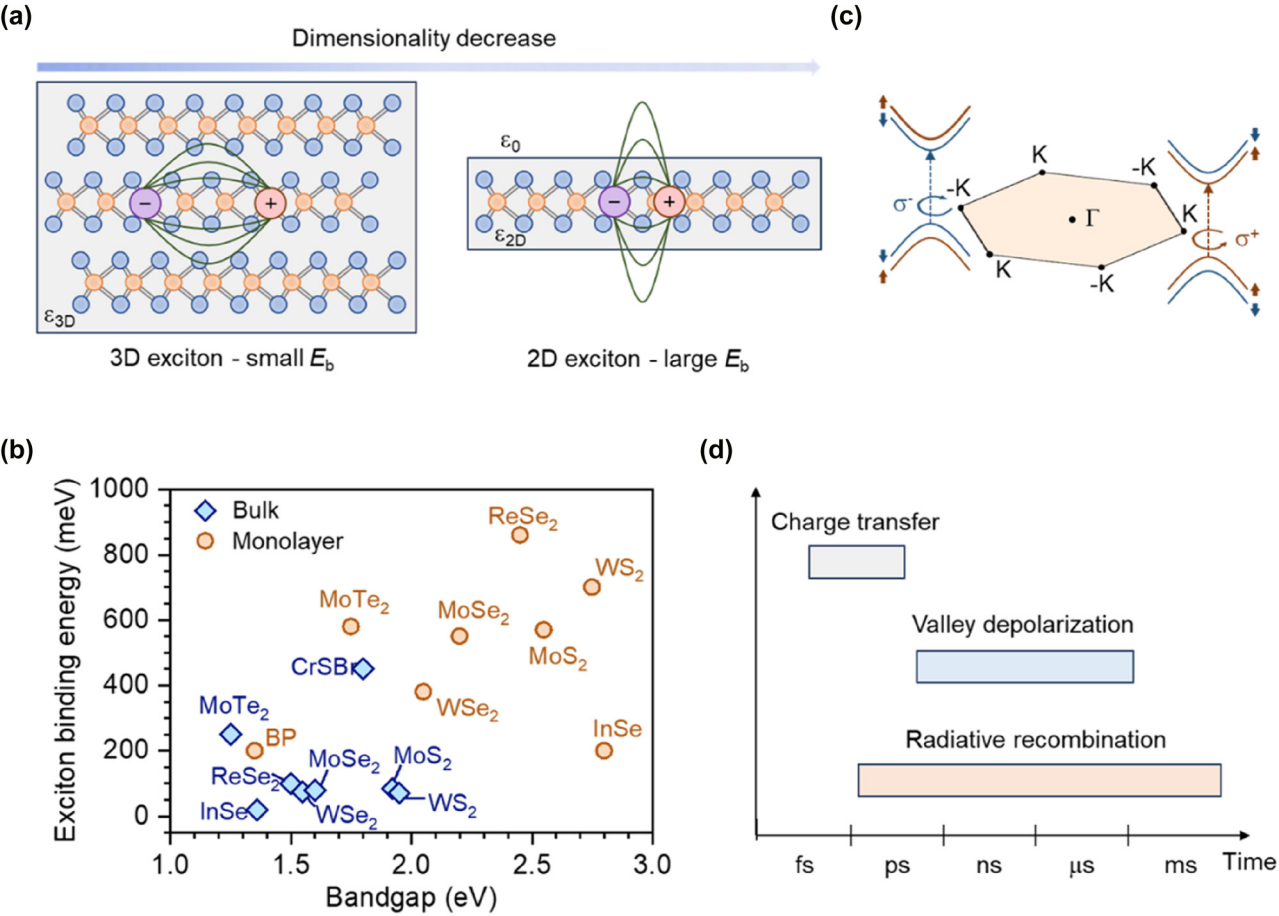
Exciton characteristics in 2D van der Waals semiconductors. (a) Schematic of electrons and holes bound into excitons in 3D systems and 2D systems. (b) Exciton binding energy *versus* bandgap for different 2D vdW semiconductors. The data are extracted from refs. [32], [33], [34], [35], [36], [37], [38], [39], [40], [41], [42], [43], [56]. (c) Schematic of bright excitons at the *K* and *K*′ corners of the Brillouin zone of a TMD monolayer, displaying spin-valley locking. (d) Time scale for charge transfer, valley polarization, and radiative recombination in 2D vdW semiconductors.

The development of novel light sources based on 2D vdW semiconductors begins with the revelation of their fundamental emission properties, which are strongly linked to their electronic and excitonic states. To achieve high brightness emission, materials with direct bandgap transitions are of primary focus. The most widely studied system is the monolayer of 2H-phase TMDs, which are stable under ambient conditions and exhibit direct bandgap characteristics at the *K* point in the Brillouin zone, with transition energies in the visible to near-infrared spectral range [10]. As the number of layers increases, interlayer coupling strengthens, shifting the valence band maximum from the *K* point to the Γ point, thereby changing the material to an indirect bandgap [3]. Because bright emission originates only from direct transitions in TMD monolayers and there is a lack of tunability in thickness, other bulk materials exhibiting direct bandgaps, such as PbI_2_, InSe, NiPS_3_, and CrSBr, have also been explored as emitters [28], [29], [30], [31]. Nonetheless, these ultrathin materials can typically be regarded as 2D systems, exhibiting characteristics distinctly different from their bulk 3D counterparts. One of the most important features is that as the material thickness decreases, the Coulomb interaction between electrons and holes is enhanced due to the weakened dielectric screening and strong geometric confinement, leading to a series of tightly bound excitonic states (Figure 1a) [2]. In 2H-phase TMD monolayers, the binding energy of 2D excitons can reach several hundred meV, approximately 30 % of the bandgap energy, significantly higher than that of traditional III–V and II–VI semiconductors, and an order of magnitude greater than that of their bulk counterparts (Figure 1b) [32], [33], [34], [35], [36], [37], [38], [39], [40], [41], [42], [43]. Additionally, some bulk materials with flat electronic bands due to lattice anisotropy (e.g., CrSBr) can also enable strongly localized excitons with a giant bulk exciton binding energy comparable to those of monolayer materials [43].

Another important feature of TMD monolayers is the spin-valley locking phenomenon. The band extrema of TMD monolayers occur at the inequivalent *K* and *K*′ points in the hexagonal Brillouin zone, where these two valleys exhibit a mirror symmetry due to time-reversal symmetry (Figure 1c) [1]. Due to the strong spin–orbit coupling in transition metal atoms, the spin states in the band structure are split between the *K* and *K*′ points, with each valley locked to opposite spin directions. As a result, TMD monolayers have two types of energy-degenerate exciton states, which are coupled to right and left circularly polarized light according to the valley optical selection rule [44], [45], [46]. This additional valley degree of freedom bridges the gap between photonics and spintronics, providing a rich physical basis for achieving novel optical, electronic, and quantum properties [47], [48].

The high spatial overlap of electron–hole wavefunctions in 2D vdW semiconductors generates a strong excitonic transition dipole moment, which means their radiative lifetimes are typically as short as 0.1–10 ps [49]. This short lifetime is not favorable for exciton accumulation and for the long-distance transfer of valley pseudospin information. Constructing 2D vdW heterostructures can overcome these limitations. Due to weak interlayer coupling and the absence of surface dangling bonds, different 2D materials can be stacked in any order without disrupting their crystal structures and can tolerate large lattice mismatches, reducing interface defects and ensuring high-quality heterostructures [6]. Atomic-level sharp type-II heterostructures support ultrafast interlayer charge transfer (sub-ps) and allow for spatial separation of electrons and holes across the two layers, thereby suppressing exciton–exciton interactions in both recombination and depolarization processes [50]. As a result, exciton recombination lifetimes can be extended to the ns to ms scale, and valley polarization lifetimes can be extended to the ns to μs scale (Figure 1d) [51], [52], [53], [54], [55].

**Figure 2:**
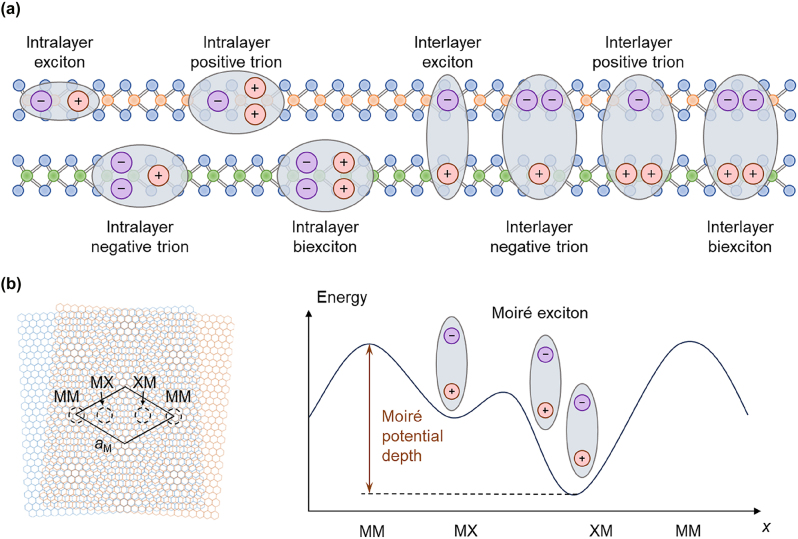
Different types of intralayer and interlayer exciton complexes. (a) Schematic of different types of intralayer and interlayer exciton complexes. (b) Schematic of a moiré superlattice formed by two hexagonal lattice with a small twist angle (left penal) and energy landscape of moiré exciton in the moiré potential (right panel).

For 2D vdW materials and their heterostructures, since bright exciton states dominate the emission process, we further introduce various types of intrinsic many-body complexes of bright excitons that emerge in these systems (Figure 2a). In fact, dark exciton states are also commonly present, and these states cannot be directly excited by light due to the requirement of spin flipping and/or phonon-assisted momentum transfer [57], [58], [59], [60]. The relative position of the dark exciton states to the optically accessible bright exciton states plays a crucial role in determining the light emission efficiency of these materials and thus technological potential. For a more detailed discussion on dark exciton states, we refer to other reviews [61], [62].

### 2.1 Neutral exciton

The basic neutral exciton consists of a negatively charged electron and a positively charged hole bound together by Coulomb forces. The electron and hole can originate from a single material, forming intralayer excitons, or can each be provided by separate layers, forming interlayer excitons [41], [51], [63]. Intralayer excitons have a higher binding energy (>500 meV) and shorter lifetimes (in the picosecond range) because the electron and hole are located within the same layer and are relatively close. In contrast, due to the spatial separation of the electron and hole, interlayer excitons have a slightly smaller binding energy (∼100–300 meV) and longer lifetime extending to the nanosecond scale. Moreover, for vertically stacked heterostructures, interlayer excitons exhibit an out-of-plane transition dipole moment, which is beneficial for creating and manipulating dipole interactions, aiding research on strongly correlated systems, developing tunable optical devices, and realizing exotic physical phenomena such as exciton flux and superfluidity [64], [65], [66]. A significant spatial overlap between the wavefunctions of intralayer and interlayer excitons will further lead to the mixing of exciton states to form hybrid excitons with optical, electrical, and dynamic properties intermediate between the two. The hybrid nature allows for the customization of the exciton oscillator strength, lifetime, and external field modulation. To facilitate the formation of hybrid excitons, the energy levels in each layer should be close and have the same spin, which has been widely observed in both homobilayer and heterobilayer systems [67], [68], [69], [70].

### 2.2 Trion

When a neutral exciton captures an additional negatively charged electron or positively charged hole, a new charged composite is formed, i.e., a negative or positive trion. The introduction of the additional carrier weakens the many-body interactions and causes the expansion of exciton wavefunction; therefore, the binding energy of trion is usually an order of magnitude lower than that of neutral exciton. The formation of intralayer and interlayer trions depends on specific environmental conditions, such as the doping level, light intensity, temperature, and applied electrical fields, which affect the free carrier density and the exciton capture ability of free carriers. Specifically, interlayer trions include two types: Type I (two identical charges in the same layer) is energetically more favorable and has been reported in most experiments [71], [72], [73], while Type II (two identical charges in different layers) has also been experimentally verified and exhibits additional polarized emission behavior due to anisotropic charge interactions [74].

### 2.3 Biexciton

Under extremely high exciton density, efficient inelastic scattering between excitons leads to the formation of a neutral complex consisting of two excitons, known as the biexciton. Intralayer biexcitons have no significant electric dipole moment, while interlayer biexcitons with charge separation occurring across the two layers possess a strong vertical electric dipole moment. Depending on the ratio of exciton spacing to the Bohr radius, biexcitons can be classified into bound and unbound types, where Coulomb attraction dominates in the former (with negative binding energy) and repulsive interactions between individual dipole excitons drive the latter (with positive binding energy [75], [76], [77], [78], [79]). Bound biexcitons are common in intralayer configurations, while unbound biexcitons are more common in interlayer configurations.

### 2.4 Moiré exciton

In vdW bilayers, when the monolayers are stacked at a small angle or with lattice mismatch, a long-period spatially periodic structure known as a moiré superlattice is formed (Figure 2b [80], [81]). The moiré superlattice introduces a periodic in-plane potential landscape at the nanoscale, which can be used to modulate the electronic band structure of the material periodically. Additionally, the periodic modulation can also arise from strain, substrate, or optical field. All excitons mentioned above can be modulated under the periodic potential of the moiré superlattice and can move in the optical lattice, forming moiré excitons when the exciton Bohr radius is smaller than the moiré period [69], [77], [82], [83], [84], [85], [86]. Moiré excitons exhibit unique optical and electronic properties, such as wavefunction localization at high-symmetry positions within the moiré superlattice, showing quasi-zero-dimensional natures with enhanced stability, flat band dispersion, and significant many-body correlations [87], [88].

**Figure 3:**
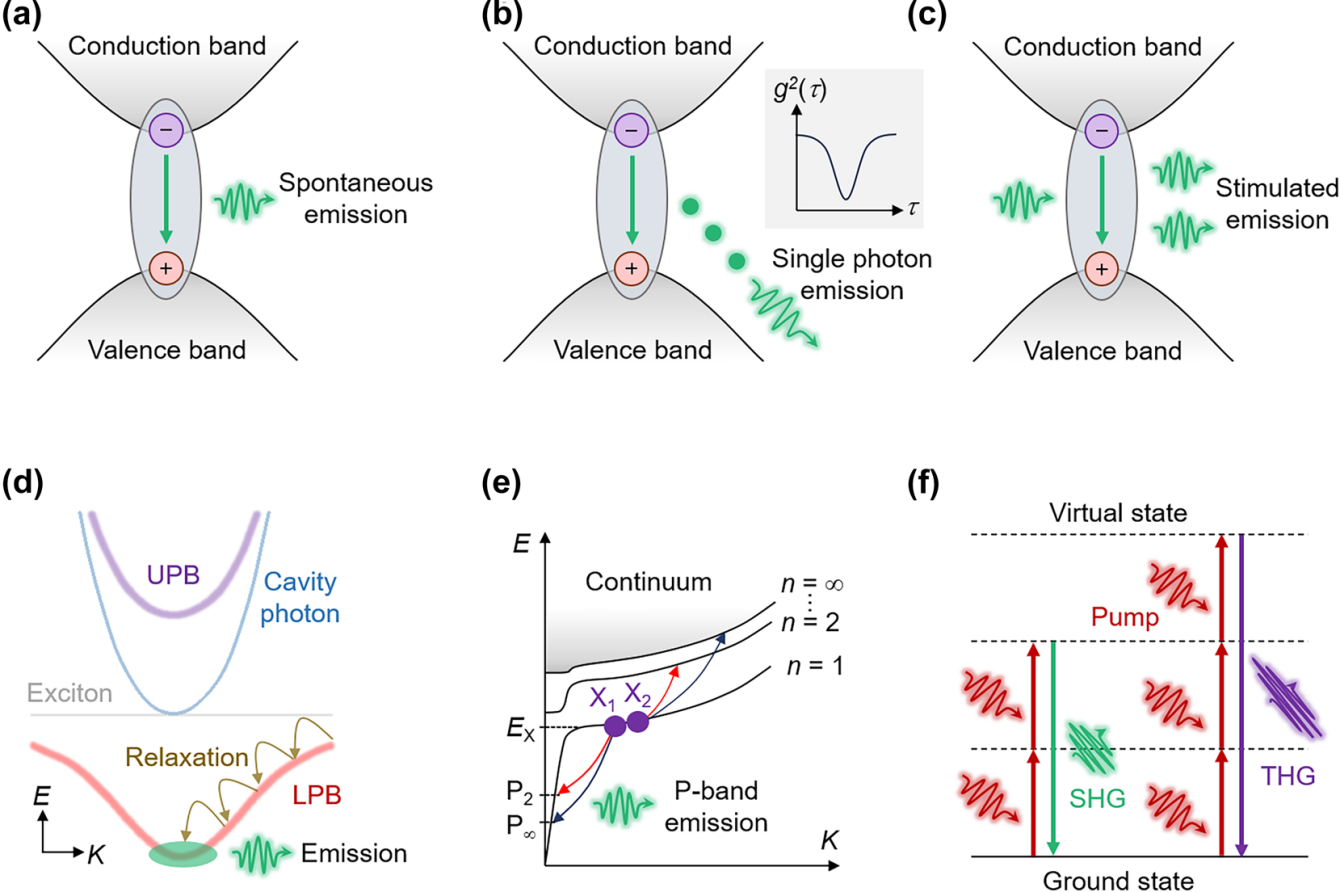
Schematic of spontaneous emission (a), SPE (b), stimulated emission (c), EP condensation (d), P-band emission (e), and second/third harmonic generation (f).

In the following sections of this review, we will discuss various emerging 2D vdW semiconductor light sources. Before delving into specific progresses, we will briefly introduce the underlying physical mechanisms associated with these light sources. The fundamental light emission in 2D vdW semiconductors is spontaneous emission (SE), where excited-state carriers, such as electrons and holes, can spontaneously recombine and release photons to return to the ground state (Figure 3a). Besides, the SPE is a special SE process, where only one photon is emitted within a specific time frame, evidenced by the intensity-correlation function at zero time delay *g* ^(2)^(0) < 0.5 (Figure 3b [20]). The SPE typically arises from localized exciton states induced by strain, electric fields, defects, or moiré periodic potentials. Single-photon sources are one of the core building blocks for photonic integrated circuits used in quantum applications, such as quantum communication and quantum computing. Excited-state carriers can be obtained through optical, electrical, or chemical excitations, and from a practical perspective, this review will focus on LEDs and electrically pumped single-photon sources that utilize electrically driven SE.

Another important light-emitting unit is the laser, which relies on stimulated emission. When an excited-state carrier is stimulated by a photon with energy matching its transition energy, the carrier will transition to the ground state and release a new photon identical to the incident photon, accompanied by light amplification (Figure 3c). Realizing a laser requires a resonant cavity to provide optical feedback, in which excitons couple with cavity photons, typically under the weak coupling regime. When the coupling rate between the excitons and cavity photons exceeds their dissipation rates, a new hybrid quasiparticle, called EP, is created in the strong coupling regime, leading to the splitting of the energy bands into upper and lower polariton branches with anticrossing behavior in energy-momentum dispersion (Figure 3d [89]). The bosonic nature of EPs allows for Bose–Einstein condensation (BEC) at high temperatures, owing to their lighter mass inherited from the photon component, with a large number of EPs occupying the ground state, resulting in photon amplification. The characteristics of EP condensation is very similar to those of conventional lasers, but without the need for population inversion, making them suitable for developing low-threshold laser-like light sources.

To bridge the gap between high-intensity/high-coherence lasers and low-intensity/low-coherence LEDs, high-intensity but low-coherence light sources are also required, which have distinct advantages in applications such as optical coherence tomography, interferometric sensing, and frequency-resolved lidar. One solution is to utilize superlinear P-band emission, which occurs through elastic exciton–exciton scattering in the presence of EPs [90]. Two excitons in the *n* = 1 exciton state can scatter, with one downward to a lower photon-like polariton state, triggering P-band emission, while the other upward to a higher excited state (*n* = 2 to ∞, Figure 3e). The final photon state for P-band emission with negligible interparticle interactions exhibits a narrow linewidth. The scattering process between exciton pairs leads to a quadratic power dependence for P-band emission, eliminating the need for population inversion, which allows for strong light output with low energy consumption.

Finally, we discuss nonlinear light sources based on 2D vdW materials, with a particular focus on the generation of higher-order optical harmonics and frequency conversion through the nonlinear polarization response of 2D vdW materials to an applied optical field (Figure 3f [91]). Compared to traditional bulk nonlinear crystals, 2D vdW materials, benefitting from their ultrathin thickness, relaxed phase matching condition, and larger nonlinear coefficient, open new opportunities for realizing miniaturized on-chip nonlinear photonic and optoelectronic devices.

## 3 2D semiconductor lasers

2D semiconductor lasers are of particular interest because of their potential applications in nanophotonic, optical communication, and integrated photonic circuits. The development of 2D semiconductor lasers is driven by the unique combination of material properties that these atomic-layer-thin materials offer, including direct bandgaps [1], [3], [10], strong excitonic effects with large exciton binding energy [2], [41], [92], spin-valley locking [44], [45], [93], [94], [95], [96], [97], [98], [99], [100], and notable charge carrier mobility [13], [101], [102], which are critical for efficient light emission and laser action.

As discussed above, monolayer TMDs exhibit a direct bandgap transition due to the absence of interlayer interactions that typically induce indirect bandgaps in bulk materials. This transition leads to a high radiative recombination quantum efficiency, which is critical for achieving the necessary population inversion in a laser system. TMDs are also distinguished by their strong excitonic effects, arising from their reduced dielectric screening and quantum confinement in two dimensions, which result in an exceptionally high exciton binding energy. This high binding energy allows excitons to remain stable even at room temperature, while also supporting a high Mott transition density (∼10^14^ cm^−2^) [10], preventing thermal dissociation into free carriers, a major advantage over conventional semiconductor lasers. Additionally, the small Bohr radius of excitons in TMDs enhances light–matter coupling, leading to strong oscillator strengths and high optical absorption coefficient, ensuring an efficient stimulated emission process. However, due to their atomically thin nature, TMD monolayers do not provide sufficient optical confinement, necessitating integration with high quality factor (*Q*-factor) optical cavities to sustain lasing. Various cavity designs, such as photonic crystal cavities (PCCs), which exploit Bragg diffraction to confine light in periodic dielectric structures, whispering-gallery-mode (WGM) resonators, which rely on total internal reflection at curved interfaces to achieve ultra-high *Q*-factor, and distributed Bragg reflectors (DBRs), which use multi-layered optical interference to enhance light feedback, all contribute to reducing the threshold carrier density required for lasing.

Unlike intralayer excitons, which form within a single monolayer and exhibit shorter lifetimes and strong recombination, interlayer excitons emerge in type-II band-aligned heterostructures (e.g., MoS_2_/WSe_2_, WSe_2_/MoSe_2_) [103], [104], where electrons and holes reside in separate layers. This spatial separation occurs due to ultrafast charge transfer (∼50 fs) following optical excitation, where electrons migrate to the conduction band of one layer while holes remain in the valence band of the other. This configuration results in several key advantages, in detail, (1) longer exciton lifetimes (∼μs), which significantly exceed those of intralayer excitons (∼ps) [54], allowing for excitonic condensation and lasing buildup; (2) a permanent out-of-plane electric dipole moment, enabling precise control over excitonic energy levels via an external electric field; and (3) reduced recombination rates, improving optical gain efficiency. To achieve interlayer exciton lasing, high-*Q* optical cavities are employed to provide sufficient optical feedback and enhance the stimulated emission process.

**Figure 4:**
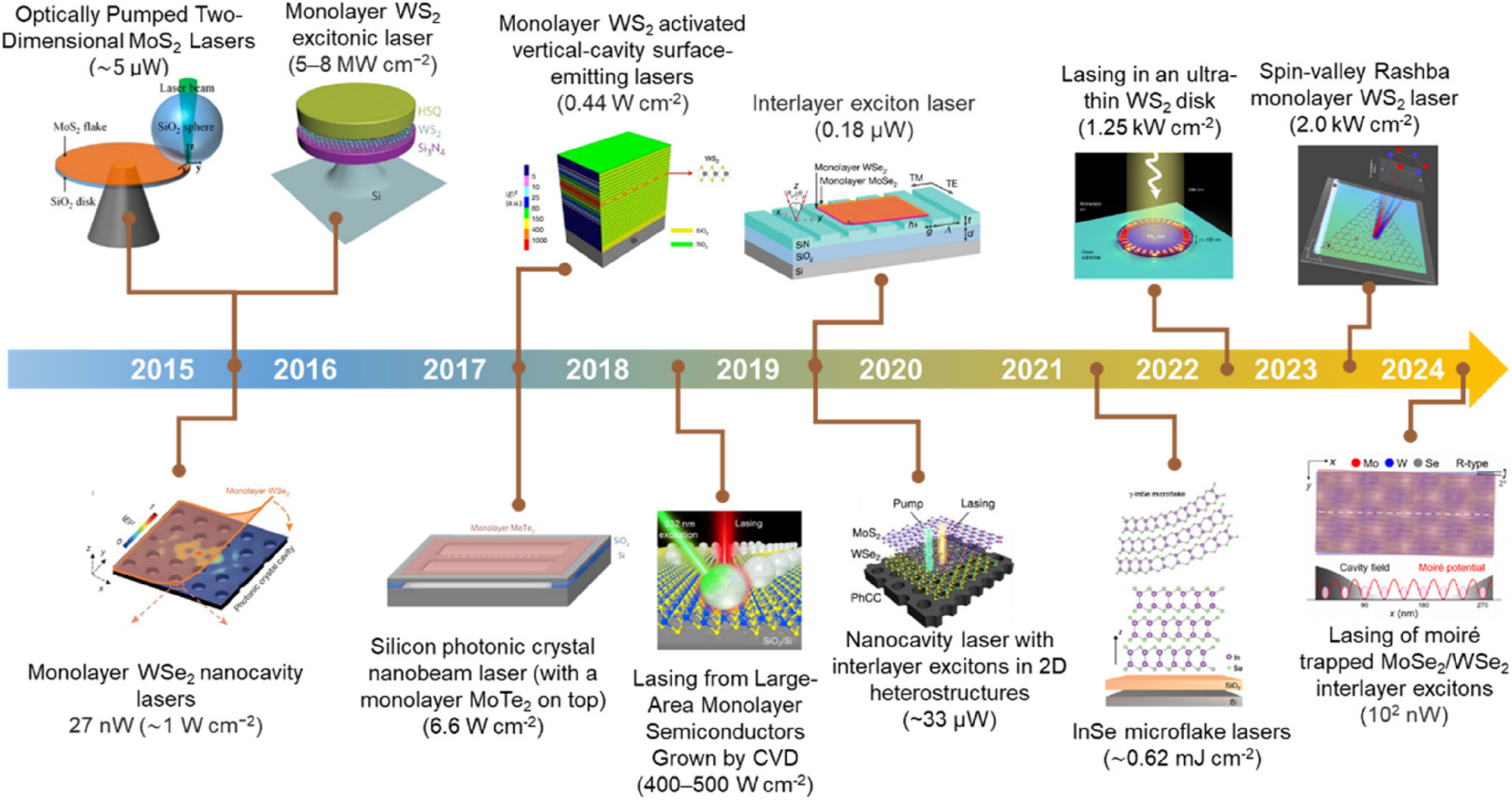
A timeline of key advancements in nanophotonics and low-power lasers based on 2D materials from 2015 to 2024. Laser systems with (intralayer exciton complexes [12], [105], [106], [107], [108], [109], [110], interlayer exciton complexes [103], [104] and moiré exciton complexes [111]) and without external cavity [29], [112].

In recent years, significant advancements in 2D semiconductor lasers have been achieved, driven by the unique properties of 2D vdW materials. Key developments include miniaturization and on-chip integration for optical communication and computing systems, room-temperature operation enhancing practical feasibility, and precise tuning of energy bands and optical properties through vdW heterostructures. Enhancements in light–matter interactions have reduced laser thresholds and increased efficiency, while the exploration of new 2D vdW materials has expanded potential applications. Integration with silicon photonics and application-driven research have further propelled the development of high-efficiency, tunable, and easily integrable lasers, profoundly impacting fields such as communication, computing, and sensing. Extensive and in-depth studies have been conducted on the lasing properties of various TMD materials integrated within different cavity structures, as well as the lasing characteristics of certain vdW materials without external cavities [12], [103], [104], [105], [106], [107], [108], [109], [110], [111], [112], as depicted in Figure 4. In this part, we introduce different 2D laser systems with and without external cavities and highlight the special features of EP in these materials.

### 3.1 Intralayer-exciton laser based on monolayer TMD with different cavities

**Figure 5:**
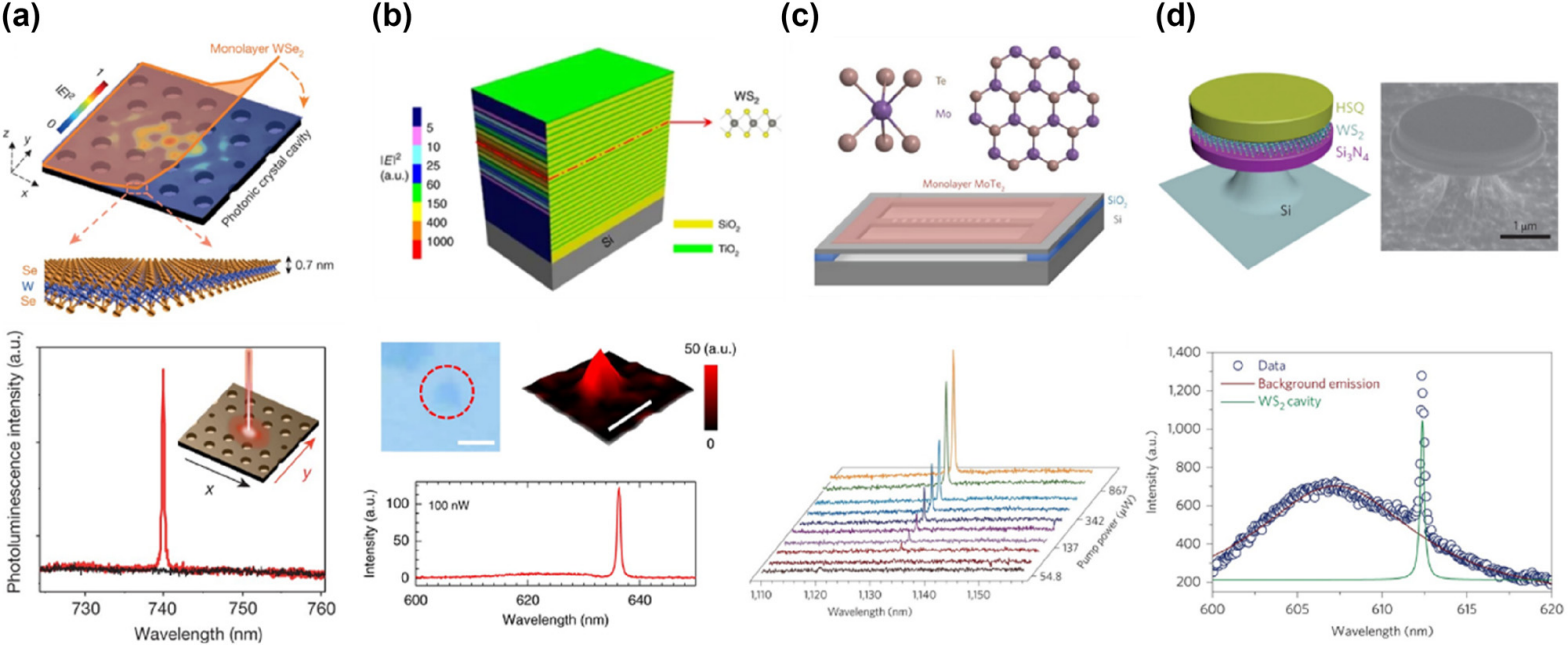
Four distinct cavity architectures for TMD-based intralayer exciton lasers. (a) Top panel: the architecture of a hybrid monolayer WSe_2_ PCC nanolaser, including a color plot depicting the electric field profile of the fundamental cavity mode (before the WSe_2_ transfer). Bottom panel: the polarization-resolved PL spectrum of the nanolaser at 80 K. The black and red lines correspond to detected linear polarizations in the *x* and *y* directions, respectively. Reproduced from ref. [12]. Copyright 2017, Springer Nature. (b) Top panel: the structural schematic of a vertical-cavity surface-emitting laser based on a monolayer of WS_2_. Middle panel: the optical image of monolayer WS_2_ (left) and the spatially resolved PL intensity mapping of the microcavity sample (right). The scale bars represent 2 µm. Bottom panel: the PL spectra collected at the center of the sample with an excitation power of 100 nW. Reproduced from ref. [108]. Copyright 2017, Springer Nature. (c) Top panel: the atomic structure of monolayer MoTe_2_ and the silicon photonic crystal nanobeam cavity structure with a monolayer of MoTe_2_ positioned on top. Bottom panel: the PL spectra at increasing pump power levels, showing the transition from spontaneous to stimulated emission. Reproduced from ref. [107]. Copyright 2017, Springer Nature. (d) Top panel: the schematic (left) and scanning electron microscope image (right) of a monolayer WS_2_ microdisk laser, comprising a sandwich structure of Si_3_N_4_/WS_2_/HSQ. Bottom panel: the PL spectrum fitted with bi-Lorentzian curves, separating the monolayer WS_2_ PL background (from the microdisk center) from the sharp cavity emission. This distinction highlights the lasing characteristics of the cavity, where the narrow emission peak signifies stimulated emission within the microdisk laser. Reproduced from ref. [106]. Copyright 2015, Springer Nature.

In 2015, Wu et al. demonstrated a continuous-wave (CW) nanolaser by integrating monolayer WSe_2_ onto a prefabricated GaP PCC (Figure 5a, top panel) [12]. The hybrid WSe_2_-PCC nanolaser showed lasing at 739.7 nm with a linewidth of 0.3 nm (Figure 5a, bottom panel), and a low lasing threshold power of 27 nW at 130 K, similar to quantum-dot PCC lasers [113]. The high initial *Q*-factor ( = 8,000) enabled a strong Purcell effect [114], [115], [116], enhancing SE and reducing the lasing threshold. After monolayer transfer, the *Q*-factor reduced to 2,500, but efficient coupling persisted with an SE coupling factor (*β*) of 0.19, demonstrating effective emission comparable to quantum-dot PCC lasers [113], [117].

The development of room-temperature, low-threshold vertical-cavity surface-emitting lasers (VCSELs) incorporating 2D semiconductor materials represents a significant step toward practical optoelectronic applications. In 2017, Yu et al. reported a room-temperature CW VCSEL using monolayer WS_2_ as the gain medium (Figure 5b, top panel) [108]. The VCSEL employed SiO_2_/TiO_2_ DBRs to form a high-reflectivity cavity. The monolayer WS_2_, positioned at the cavity’s antinode, maximized light–matter interaction and SE enhancement via the Purcell effect. The laser achieved a low threshold power of 5 nW at 636.3 nm (Figure 5b, bottom panel), with a *Q*-factor of 640.

Silicon’s bandgap (∼1.12 eV, 1,100 nm) causes high absorption for emissions above this wavelength [118], [119], making most TMDs unsuitable for integration with silicon cavities. MoTe_2_, with a bandgap greater than 1.7 eV and a PL peak at 1.1 eV, is ideal [39], [120], [121]. In 2017, Li et al. demonstrated CW lasing using monolayer MoTe_2_ integrated with a silicon nanobeam cavity (Figure 5c, top panel) [107]. The cavity achieved a *Q*-factor of 5,603, with lasing at 1,132 nm (linewidth: 0.202 nm, bottom panel of Figure 5c) and a low threshold power density of 6.6 W/cm^2^, which is notably lower than other excitonic lasers in ultraviolet wavelengths operating at room temperature [122]. This design allows efficient lasing at wavelengths where silicon is transparent, making it promising for silicon photonics.

Microdisk resonators are crucial for 2D lasers, supporting WGMs that confine light efficiently [123]. Ye et al. integrated monolayer WS_2_ with a microdisk resonator in 2015, creating a high-quality WGM cavity with a *Q*-factor of 2,604 (Figure 5d, top panel) [106]. The lasing mode at 612.2 nm (Figure 5d, bottom panel), along with additional modes, demonstrated efficient optical confinement with a lasing threshold between 5 and 8 MW/cm^2^. Linewidth narrowing from 0.28 nm to 0.24 nm indicated the lasing onset, and this work demonstrated the potential for valley-selective lasing, offering new functionalities in 2D vdW material-based photonics [94].

**Figure 6:**
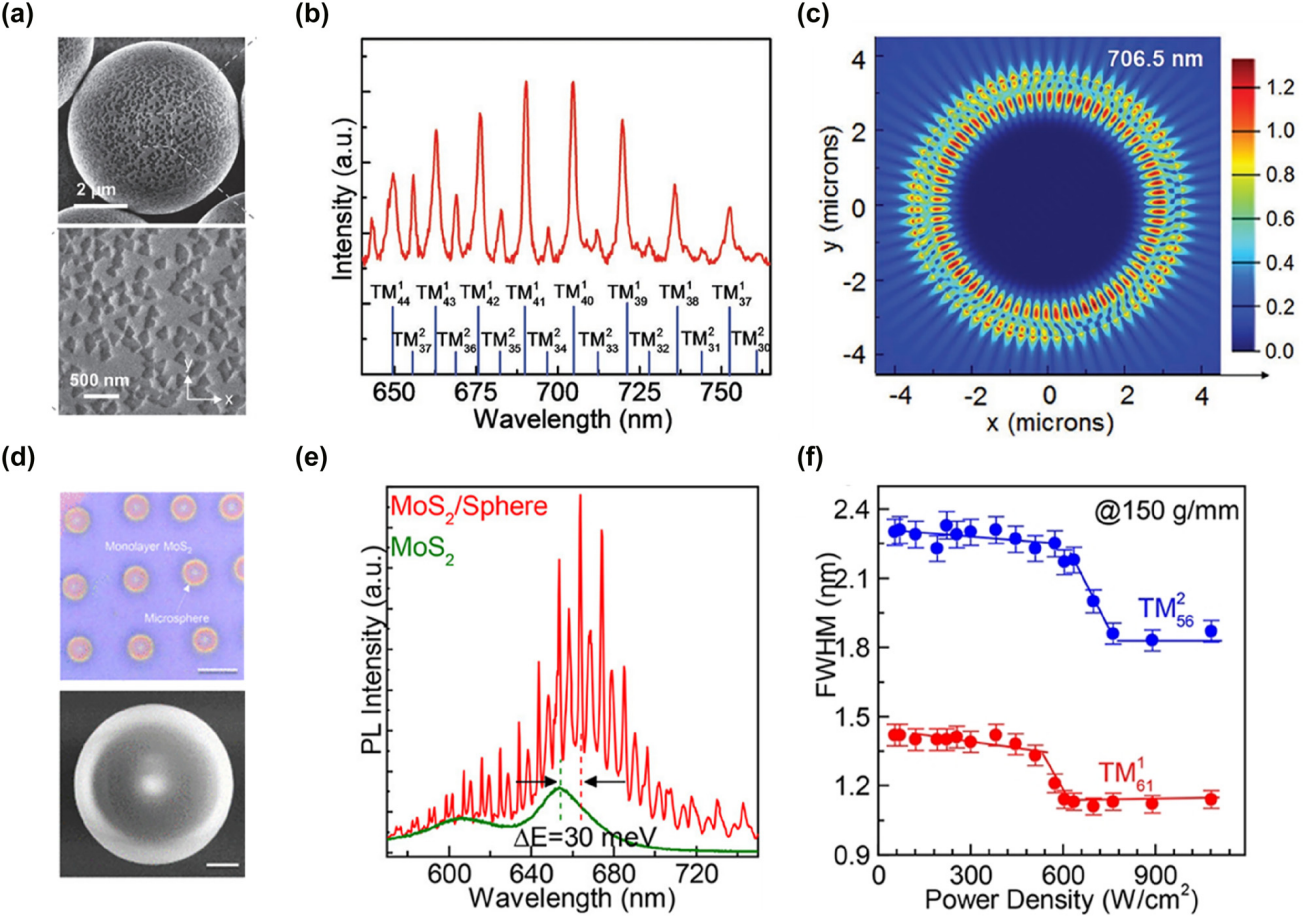
Application of SiO_2_ microspheres on 2D TMD materials emission. (a) Top-view scanning electron microscope images of monolayer MoS_2_ grown on SiO_2_ microspheres at different magnifications. (b) PL spectrum of MoS_2_ on a single SiO_2_ microsphere, with background emission subtracted to clearly display the WGM resonance peaks. (c) A FDTD simulation, showing the electric field distribution pattern of a transverse magnetic (TM) mode at a resonance wavelength of 706.5 nm in the microcavity. Panel (a–c): reproduced from ref. [128]. Copyright 2017, Wiley-VCH. (d) Upper panel: the optical image of the MoS_2_/microsphere array structure. Lower panel: the scanning electron microscope image of a single SiO_2_ microsphere within the array. (e) The PL spectra comparing MoS_2_/microsphere (red line) with monolayer MoS_2_ on a SiO_2_–Si substrate (olive line) at room temperature. The higher PL intensity of the MoS_2_/microsphere configuration indicates enhanced emission due to the lensing effect of the microsphere, which focuses excitation light onto the MoS_2_ layer more efficiently. (f) The FWHM values for the TM61^1^ and TM56^2^ modes of the WGM laser as a function of excitation power. The narrowing of the FWHM at higher excitation powers demonstrates the threshold behavior typical of lasing, confirming the WGM laser operation in the MoS_2_/microsphere system. Panel (d–f): reproduced from ref. [109]. Copyright 2018, American Chemical Society.

Tuning the optical properties of 2D semiconductors is crucial for developing high-performance photonic devices. WGM cavities, with their high *Q*-factor, significantly enhance light–matter interactions, making them ideal for optical amplification and sensing [124], [125], [126], [127]. Mi et al. used chemical vapor deposition (CVD) to deposit monolayer MoS_2_ onto SiO_2_ microspheres, forming MoS_2_/SiO_2_ microcavities (Figure 6a) [128]. At room temperature, multiple WGM peaks were observed between 650 and 750 nm under CW excitation (Figure 6b), and these peaks were validated through finite-difference time-domain (FDTD) simulations (Figure 6c). These microcavities exhibited refractive index sensing with a sensitivity of 150 nm per refractive index unit, highlighting their potential for optoelectronic sensors. Adjusting the microsphere diameter allowed further tuning of WGM modes to optimize sensing performance.

Traditional 2D vdW material lasers are typically fabricated via mechanical exfoliation, which poses challenges for reproducibility and large-scale production. To overcome this, Zhao et al. grew large-area MoS_2_ films using CVD and coupled them with SiO_2_ microspheres to form WGM cavities (Figure 6d) [109]. The microspheres reduced screening effects, enhancing carrier localization and improving effective optical gain [129], [130]. This setup increased exciton SE efficiency (Figure 6e) and enabled strong CW lasing output over a wide temperature range (77–400 K). The devices exhibited low lasing thresholds (32–580 W/cm^2^), which is much lower than that of many traditional laser structures [105], [106], [107], and the full width at half maximum of the lasing mode significantly narrowed beyond the threshold (Figure 6f), indicating strong optical gain and stable lasing. These findings demonstrate the potential of microsphere-coupled MoS_2_ microcavities for developing low-power, wide-temperature-range optical devices.

### 3.2 Interlayer-exciton laser based on TMD heterobilayer

Previous research has focused on nonlinear lasing behavior and linewidth narrowing in monolayer TMDs [12], [105], [106], [107], [108], [109]. However, the spatial coherence of the emission has remained unexplored, and the photon flux was observed to be below the stimulated emission threshold, making it complex to rule out localized excitons as the origin of the lasing [131]. Meanwhile, monolayer excitons are limited by their intrinsic band structure, resulting in lacking electrical tunability. In contrast, interlayer excitons are electrically tunable, and external electric fields can adjust the dipole strength and exciton–photon interaction, making bilayer devices advantageous for dynamic modulation and integrated photonics applications.

**Figure 7:**
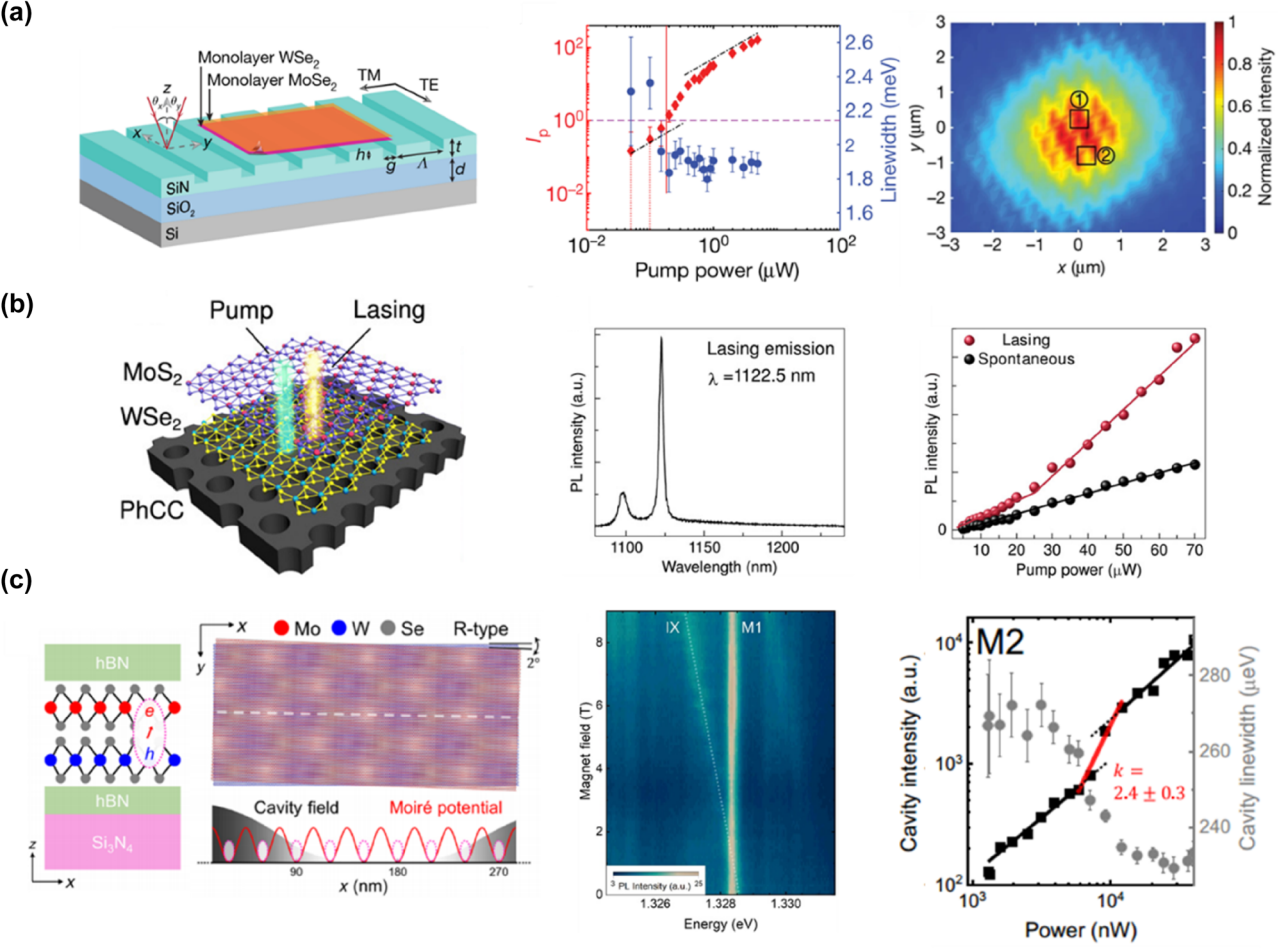
Three distinct cavity structures for TMD-based interlayer exciton lasers. (a) Left panel: the schematic of a heterobilayer WSe_2_–MoSe_2_ laser device integrated with a SiN grating cavity. Middle panel: a plot of the photon occupancy and linewidth of the TE-polarized emission as a function of pump power. The photon occupancy (red dots) shows a superlinear increase at the threshold (vertical red line), while the linewidth (blue dots) narrows, indicating the onset of lasing. Right panel: the interference pattern observed in the Michelson interferometer setup at a power above the lasing threshold (20 μW). Reproduced from ref. [104]. Copyright 2019, Springer Nature. (b) Left panel: schematic of a nanocavity laser device using a MoS_2_/WSe_2_ heterostructure as the gain medium. Middle panel: the emission spectrum of the cavity lasing mode at 5 K with a CW pump power of 190 mW. The lasing action is observed at approximately 1,122.5 nm with a linewidth of ∼2.7 nm. Right panel: the L–L curve for the laser. The cavity mode intensity (red dots) exhibits a clear kink, typical of lasing onset, while the background emission (black dots) remains linear. Reproduced from ref. [103]. Copyright 2019, AAAS. (c) Left panel: schematic of the MoSe_2_/WSe_2_ heterobilayer integrated with a nanocavity and encapsulated by hexagonal boron nitride (hBN). The right side displays the electric field distribution in the nanocavity. Middle panel: PL spectrum for the MoSe_2_/WSe_2_ heterobilayer coupled with a nanocavity, recorded under low excitation power at 88 nW and in the presence of a magnetic field. Right panel: power-dependent behavior of the PL intensity and linewidth of cavity mode M2, indicating the transition to lasing. Reproduced from ref. [111]. Copyright 2024, AAAS.

To realize interlayer exciton lasing, Paik et al. used a rotationally aligned WSe_2_–MoSe_2_ heterobilayer integrated with a SiN grating resonator (Figure 7a, left panel) [104]. This heterobilayer served as the gain medium, where interlayer excitons formed by electrons and holes in different monolayers enabled lasing. The alignment created a direct bandgap between the *K* valleys of the two monolayers, enhancing oscillator strength and efficient carrier transfer [132]. The resonator was designed to match exciton resonance, supporting cavity modes overlapping with the heterobilayer and providing spatial coherence [133]. Power-dependent PL measurements showed a linear increase in emission intensity above the threshold, confirming the onset of stimulated emission (Figure 4a, middle panel). Coherence measurements using a Michelson interferometer confirmed spatial coherence across the emission region (Figure 7a, right panel). Compared to monolayer exciton lasers, this system benefits from electrically tunable dipole interactions, robust valley polarization, and efficient population inversion [132], [134], [135], [136].

Unlike many interlayer exciton lasers requiring cryogenic temperatures [104], [111], Liu et al. demonstrated room-temperature lasing by integrating a MoS_2_/WSe_2_ heterobilayer with a PCC in 2019 (Figure 7b, left panel) [103]. The interlayer excitons emitted in the infrared range (1,122.5 nm), making them compatible with the larger bandgap of silicon (Figure 7b, middle panel). The lasing threshold was approximately 33 mW (Figure 7b, right panel), and the longer lifetime of interlayer excitons allowed for lasing with lower *Q*-factors, suitable for practical integrated photonic devices [137], [138].

Moiré excitons also play a significant role in interlayer systems. These excitons are formed when two slightly misaligned 2D vdW materials create a moiré superlattice, providing periodic confinement potential [84], [86], [139], [140], [141]. Qian et al. demonstrated lasing from moiré excitons in a WSe_2_–MoSe_2_ heterobilayer encapsulated in hBN and coupled with a high-*Q* PCC (Figure 7c, left panel) [111]. Strong coupling between the cavity mode and excitons was observed, evidenced by a linear Zeeman shift under an applied magnetic field and linewidth narrowing above the lasing threshold (Figure 7c, middle panel and right panel), indicating increased coherence. These results highlight the tunability of interlayer exciton-cavity coupling, opening possibilities for quantum light sources and nanophotonic devices [142].

### 3.3 2D vdW materials lasers without external cavities

The previously discussed 2D semiconductor lasers present benefits such as small size, low lasing thresholds, and tunable emission. However, they have the drawbacks of limited controllability, high optical losses on silicon substrates, and the complexity of fabricating external optical cavities. To reduce the production cost of on-chip integrated laser devices, simpler and more efficient large-scale manufacturing processes are crucial. In contrast, InSe demonstrates excellent compatibility with silicon and the ability to act as both the gain medium and the optical resonator [143], [144], [145], [146], [147], achieving lasing without the need for external optical cavities. This unique capability makes InSe a highly promising, cost-effective solution for integrated on-chip lasers.

**Figure 8:**
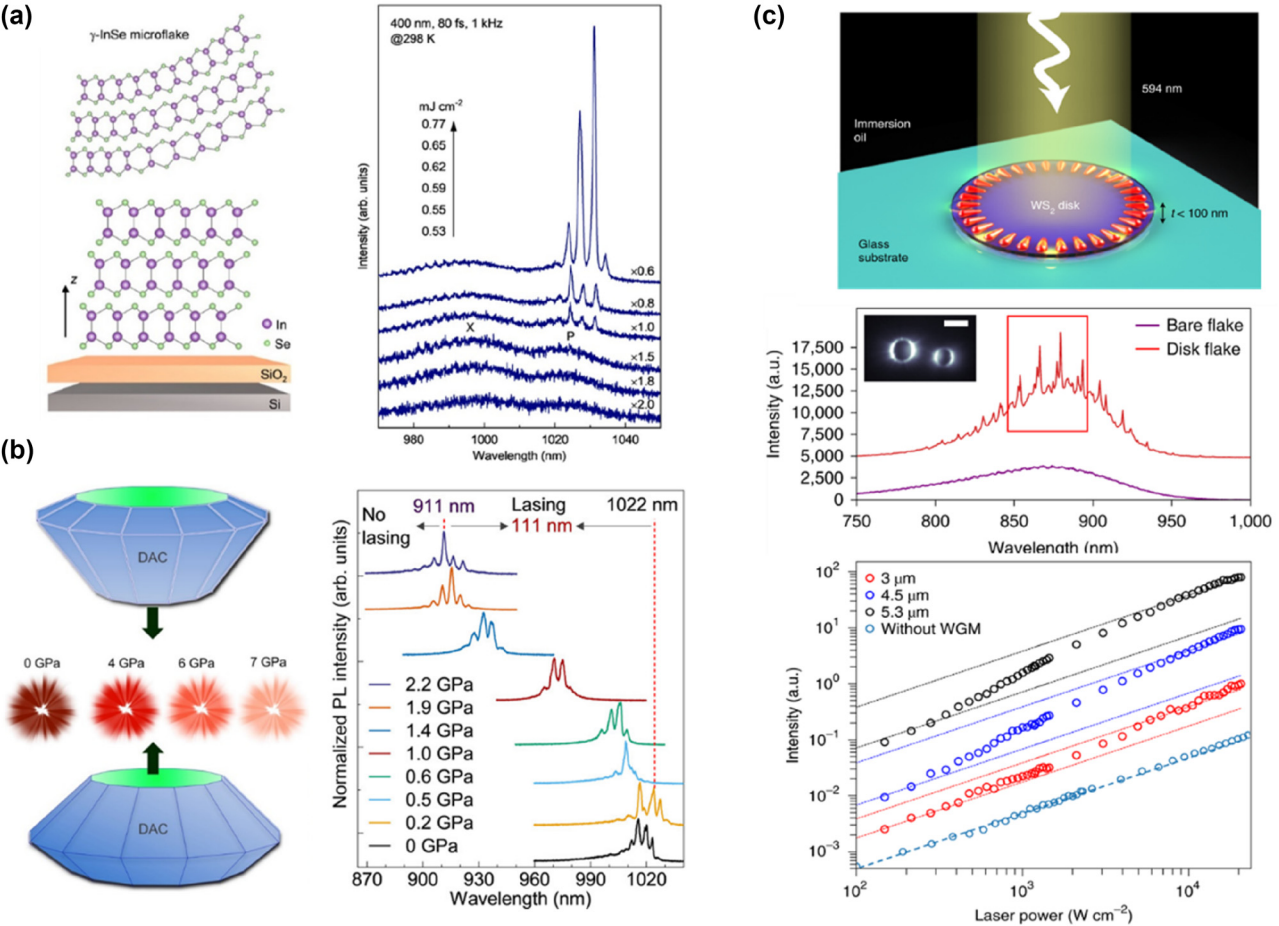
Laser emission and modulation of vdW materials without external optical cavities. (a) Left panel: schematic of mechanically exfoliated γ-InSe microflake placed on a SiO_2_/Si substrate. Right panel: the power-dependent PL spectra of a single InSe microflake at 298 K under femtosecond pulse laser excitation (400 nm, 1 kHz, 80 fs). Reproduced from ref. [29]. Copyright 2021, American Chemical Society. (b) Left panel: schematic of a diamond anvil cell used for high-pressure experiments. Right panel: the laser PL spectra of a single γ-InSe platelet under hydrostatic pressure ranging from 0 to 2.2 GPa. During the application of pressure, the laser emission’s central wavelength shifts from 1,022 nm to 911 nm. Reproduced from ref. [151]. Copyright 2022, American Chemical Society. (c) Top panel: 3D schematic image of an optically pumped WS_2_ disk nanolaser. Middle panel: the PL spectrum for a patterned WS_2_ disk, revealing multiple WGM peaks on top of a broad emission background from the indirect bandgap transition. The inset displays an emission image of the WS_2_ disk at the lasing wavelength, above the lasing threshold, which shows the light emission from the disk edge where the WGMs are formed. Bottom panel: the L–L curves of WS_2_ microdisks with different diameters. The green data indicate the SE from the indirect bandgap without coupling to WGMs, while the WGM-associated lasing action shows a nonlinear increase in intensity, indicating lasing behavior. Reproduced from ref. [112]. Copyright 2022, Springer Nature.

In 2021, Li et al. mechanically exfoliated InSe microflakes of varying thicknesses and used optical pumping to achieve room-temperature near-infrared lasing [29]. InSe, a vdW crystal with covalently bonded Se–In–In–Se layers was exfoliated to form smooth surfaces (Figure 8a, left panel). Power-dependent PL spectra of InSe microflakes (Figure 8a, right panel) showed a transition from SE to lasing, with two peaks at 995 nm (X-peak, attributed to exciton recombination) and 1,027 nm (P-band, attributed to exciton–exciton scattering) [144]. At higher excitation power (0.62 mJ/cm^2^), multiple narrow lasing peaks appeared above the P-band with a free spectral range of ∼3.38 nm and FWHM of 1.02 nm. Laser-printed microdisks (30 μm diameter) had reduced thresholds (∼0.53 mJ/cm^2^) compared to pristine microflakes, with emission coupled out from the microdisk’s edge, indicating in-plane WGM resonance [148]. These results highlight InSe’s potential for near-infrared on-chip lasers for imaging, sensing, and optical interconnects [89], [138], [149], [150]. To expand InSe’s emission characteristics, Zhao et al. applied hydrostatic pressure, achieving broad spectral tuning (Figure 8b) and demonstrating the flexibility of InSe for tunable near-infrared microlasers [151].

In studies of 2D TMD lasers, monolayer materials are often used due to their direct bandgap, which allows efficient radiative recombination without requiring phonons [1], [3], [10]. However, large-scale fabrication of monolayers is challenging. Achieving lasing in nonmonolayer TMDs with indirect bandgaps would simplify mass production of 2D semiconductor lasers. In 2022, Sung et al. demonstrated that an ultra-thin WS_2_ disk (∼50 nm) supports WGMs and provides sufficient optical gain for lasing without an external cavity (Figure 8c, top panel) [112]. The WS_2_ disk, fabricated via mechanical exfoliation and reactive ion etching, served as both gain medium and resonant cavity. Its emission spectrum showed sharp WGM peaks over a broad indirect-bandgap background (Figure 8c, middle panel). With increased pumping power, the WGM peaks transitioned from SE to amplified spontaneous emission (ASE) and eventually to lasing, forming an “S-shape” in the L–L curve (Figure 8c, bottom panel). The lasing relied on phonon-assisted emission through a three-level system. This work challenges the belief that indirect-bandgap materials are unsuitable for lasing and underscores their potential for optical and optoelectronic applications.

### 3.4 A special type: emission from EP

The above discussion focused on exciton lasers in 2D semiconductor materials, which rely on stimulated emission of photons to generate coherent light, requiring a high density of photons to achieve population inversion. Next, we introduce a special and efficient form of light–matter interaction – EPs. EPs are hybrid quasi-particles formed through the strong coupling between photons and excitons, combining the light-like properties of photons with the matter-like properties of excitons [152]. EPs are unique in that they combine the low effective mass of photons with the strong interaction properties of excitons. Due to the photonic component, polaritons can propagate with an extremely low effective mass [153], resulting in high mobility, making them ideal for fast-response optical devices. Meanwhile, the excitonic component imparts strong nonlinearity [154], which makes EPs particularly advantageous for achieving significant nonlinear optical effects at low power. This strong light–matter coupling results in Rabi splitting, leading to the formation of two polariton branches – the upper polariton branch (UPB) and the lower polariton branch (LPB) – characterized by distinct energy levels and hybrid properties. In the dispersion relation, an anticrossing phenomenon between exciton and photon energies can be observed from angle-resolved spectroscopy, which demonstrates the occurrence of strong coupling between the two, leading to the formation of EPs [155].

### 3.5 Emission from EP with external cavities

**Figure 9:**
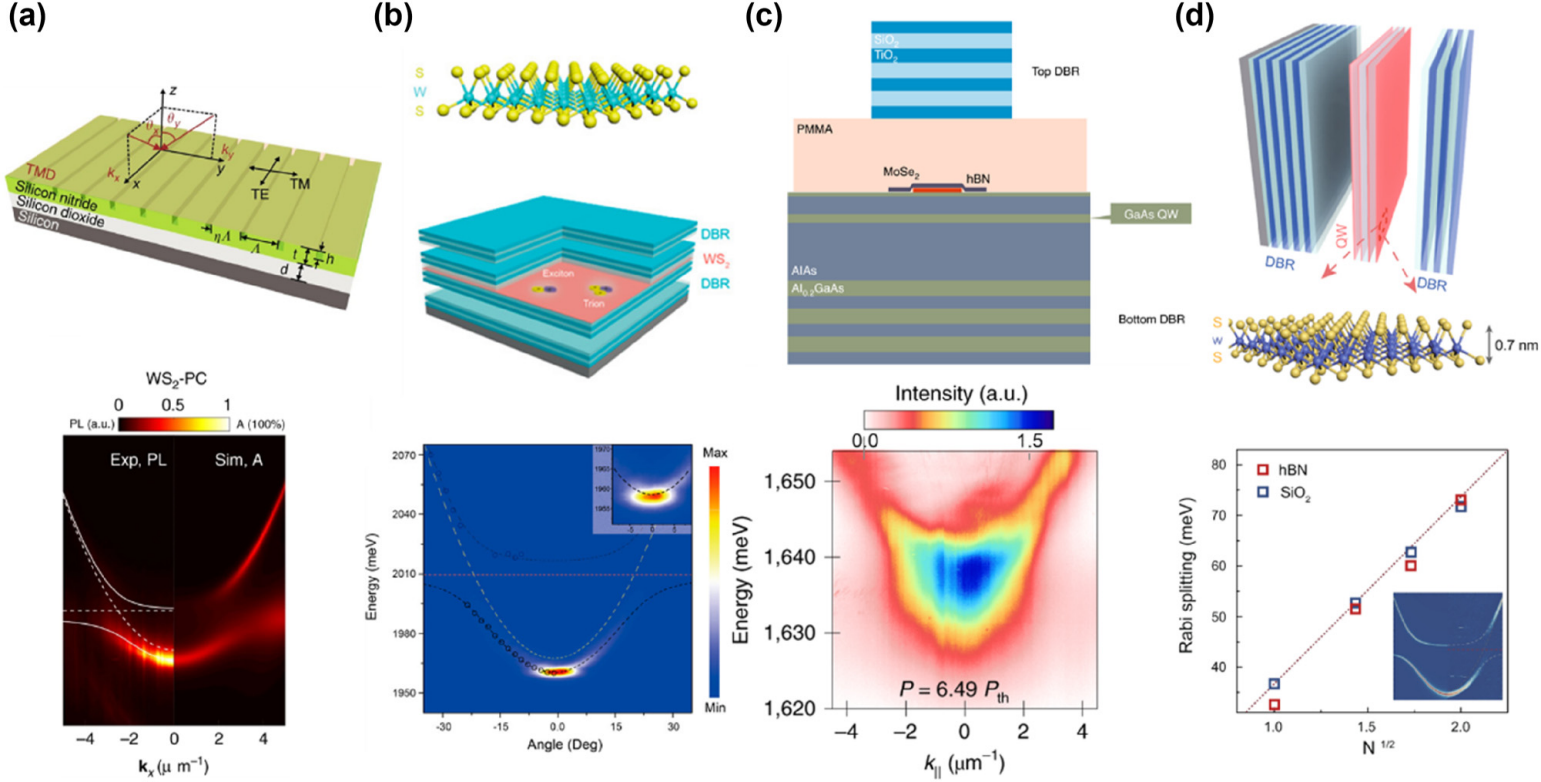
Emission from EP with external cavities. (a) Top panel: schematic of the structure integrating a monolayer TMD, such as WSe_2_ or WS_2_, with a 1D photonic crystal (PC). Bottom panel: angle-resolved PL map and corresponding simulated absorption spectra of the WS_2_-PC integrated device at room temperature. Reproduced from ref. [133]. Copyright 2018, Springer Nature. (b) Top panel: schematic of the structure integrating a monolayer WS_2_ into an all-dielectric *λ*/2 planar microcavity. Bottom panel: the angle-resolved PL map of the WS_2_ microcavity above the lasing threshold. The inset provides a zoomed-in view of the ground state, showing that the intense ground state emission is associated with the formation of a localized polariton condensate in a spatial trap. Reproduced from ref. [163]. Copyright 2021, American Chemical Society. (c) Top panel: schematic of the sample structure where a monolayer MoSe_2_ is embedded in a hybrid III/V dielectric microcavity. The structure consists of DBR, hBN, and polymethyl methacrylate spacer layers. Bottom panel: the dispersion relation map of EP condensation at pump power above the threshold (*P* = 6.49 P_th_). Reproduced from ref. [169]. Copyright 2023, Springer Nature. (d) Top panel: schematic diagram of the WS_2_ superlattice embedded in a full dielectric planar microcavity. Bottom panel: the relationship between the Rabi splitting and the square root of the number of layers, comparing the experimental results using hBN (red) and SiO_2_ (blue) as insulators. It is observed that the Rabi splitting increases with the square root of the number of layers, demonstrating the enhancement of coupling strength in the multilayer WS_2_ superlattice. The inset shows the angle-resolved reflectivity map for a microcavity containing a WS_2_ superlattice with two layers. Reproduced from ref. [170]. Copyright 2023, Springer Nature.

Zhang et al. developed a novel strong coupling system combining one-dimensional (1D) photonic crystals (PCs) with monolayer TMDs like WSe_2_ and WS_2_ (Figure 9a, top panel) [133]. The SiN PCs coupled with the monolayer TMDs provided a compact platform for strong coupling at room temperature, with observed mode anticrossing (Figure 9a, bottom panel) and tunable Fano resonances. Adjusting PC parameters allowed flexible control over polariton modes, suggesting applications in polariton lasers, amplifiers, and switches [156], [157], [158], [159], [160].

As bosons, EPs have the ability to undergo BEC under appropriate conditions. The process of BEC can be summarized as generation of EPs, cooling and thermalization, stimulated scattering leading to accumulation in the ground state, and finally formation of a macroscopic coherent state. In this condensed state, the coherence of polaritons results in the emission of coherent light, which manifests as ultra-low threshold lasing [153], [154], [155], [161]. However, traditional polariton systems, such as those based on III–V materials (e.g., GaAs [162]), typically require cryogenic temperatures to achieve condensation. Zhao et al. demonstrated room-temperature polariton condensation in a monolayer WS_2_ microcavity using a *λ*/2 structure embedded between DBRs (Figure 9b, top panel) [163]. The above-threshold angle-resolved PL map displays a sharp and intense peak near the ground state, which does not fully follow the LPB dispersion indicated by the black dashed line but instead appears delocalized in momentum space, suggesting the formation of a localized polariton condensate (Figure 9b, bottom panel). Meanwhile, spatial traps increased local polariton density, promoting condensation. This work suggests potential in valleytronics, quantum information, and low-power coherent light sources [156], [164], [165], [166], [167], [168]. Solanas et al. reported bosonic condensation of EPs in a microcavity loaded with a monolayer of MoSe_2_ at cryogenic temperatures (Figure 9c) [169]. Under an external magnetic field, valley polarization was observed, with an energy splitting between *K* and *K*′ polaritons. This demonstrates potential for valleytronic optoelectronic devices.

TMD materials can be stacked to form artificial vdW superlattices because the weak vdW forces between layers allow precise control over the stacking sequence and alignment, providing unique opportunities to manipulate light–matter interactions. Gaining insight into these interactions enables precise control over quasiparticles that merge the properties of light and matter. Zhao et al. demonstrated control of coupling strength by embedding multiple WS_2_ monolayers in a planar microcavity (Figure 9d, top panel) [170]. Increasing the number of layers enhanced vacuum Rabi splitting from 36 meV to 72 meV (Figure 9d, bottom panel), improving EP stability. Additionally, phase space filling effects and long-lived dark excitons were observed, paving the way for low-power optical circuits and applications in photonics, quantum information, and integrated systems [171].

### 3.6 Emission from EP without external cavities

As stated above, EPs have traditionally been realized using external optical cavities such as Fabry–Pérot resonators, DBRs, or plasmonic nanostructures. These cavity-based systems enhance exciton–photon interactions by providing strong optical confinement, resulting in large Rabi splitting and enabling applications in polariton lasing, quantum optics, and nonlinear photonics. However, such architectures come with practical limitations, including fabrication complexity, integration challenges, and scalability issues, particularly for ultra-thin, atomically layered materials like TMDs. Recently, a new class of EPs has emerged, which does not require external cavities. Instead, these self-hybridized EPs rely on intrinsic optical resonances within the TMD itself or engineered photonic structures such as gratings [172] and PCs [173]. By leveraging the high refractive index, strong excitonic response, and in-plane light confinement of TMD multilayers, researchers have demonstrated cavity-free EPs in bare WS_2_ layers [174] and WS_2_-based nanostructures [172], [173]. These advancements pave the way for compact, highly tunable polaritonic devices that can operate at room temperature without the constraints of external cavity fabrication. The following discussion explores three studies that demonstrate EP emission in cavity-free systems.

**Figure 10:**
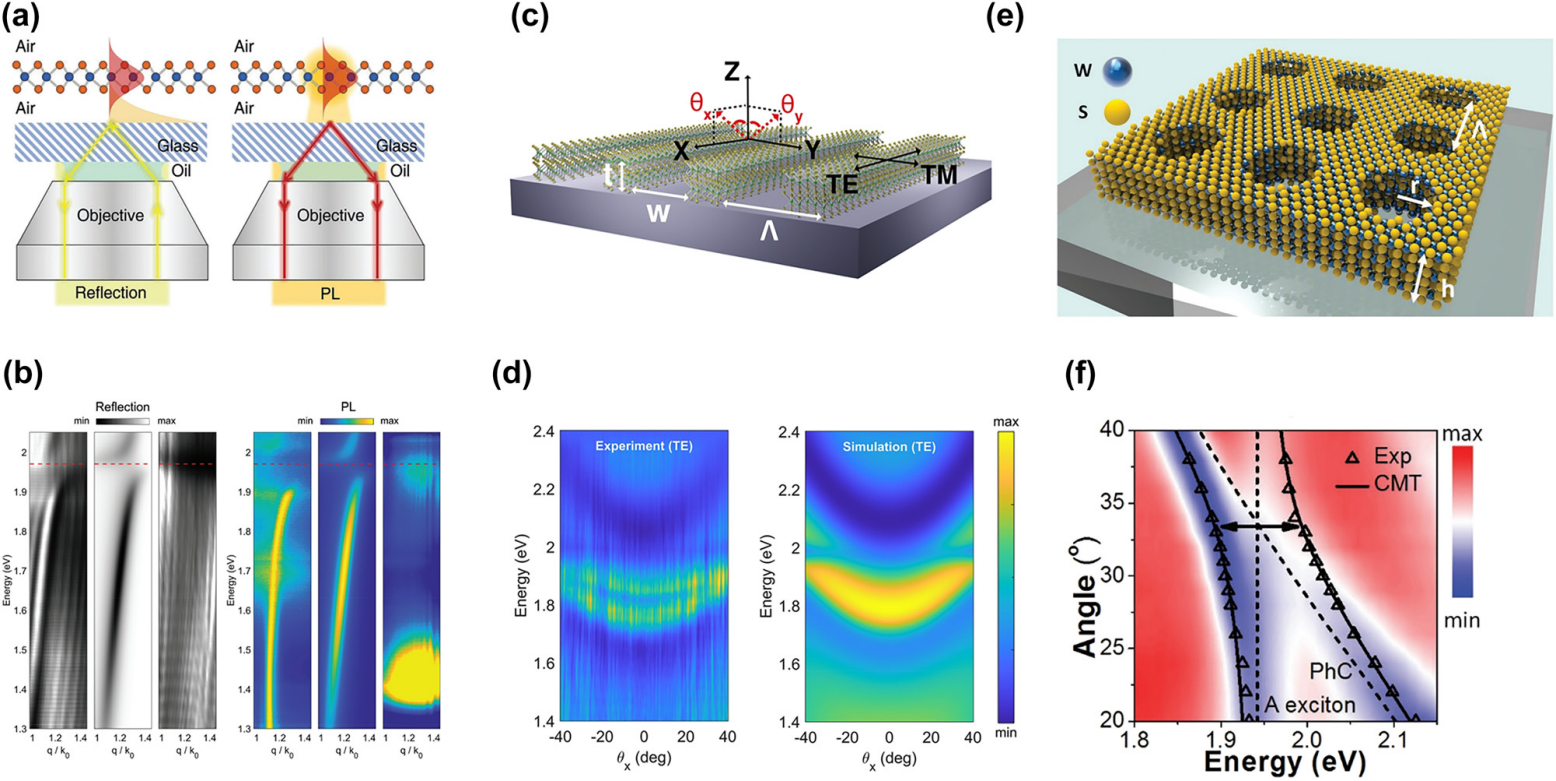
Emission from EP without external cavities. (a) Schematic of evanescent field coupling. Left panel: white-light reflection measurements probe photon modes in suspended WS_2_. Right panel: PL measurement under 594 nm laser excitation. (b) Angle-resolved reflection and PL spectra. Left (right) panel: angle-resolved reflection (PL) spectra for suspended WS_2_ layers (left), simulation (middle), and nonsuspended WS_2_ layers (right). A clear anticrossing behavior is visible, confirming strong exciton–photon coupling, leading to the formation of EP branches. Panel (a–b): reproduced from ref. [174]. Copyright 2022, Wiley-VCH. (c) Schematic of the 1D WS_2_ grating structure. (d) Left (right) panel: experimentally measured (theoretical) TE-polarized angle-resolved reflection spectrum in *x*-direction. A strong anticrossing behavior appears near 1.97 eV (exciton resonance). Panel (c–d): reproduced from ref. [172]. Copyright 2023, De Gruyter. (e) The structural design of an ultrathin WS_2_ PC. A patterned square array of air holes is fabricated in the layered WS_2_ on a glass substrate. (f) The experimental angle-resolved transmission spectrum of the WS_2_ PC. Two clear polariton branches are observed, confirming strong exciton–photon coupling. Panel (e–f): reproduced from ref. [173]. Copyright 2020, Wiley-VCH.

In 2022, Shin et al. provided direct experimental confirmation of self-hybridized EPs in bare WS_2_ multilayers, proving that strong exciton–photon coupling can occur without external optical cavities [174]. Using evanescent field coupling (Figure 10a), the authors investigated the dispersion, tunability, and valley polarization of these guided EPs. Their findings reveal clear anticrossing behavior near the exciton resonance (Figure 10b), with Rabi splitting energy varying based on layer thickness, confirming thickness-dependent strong coupling effects. Additionally, they demonstrated that the guided EPs retain valley polarization up to 0.2 at room temperature, making them promising candidates for valleytronic applications. Furthermore, they showed that the EP dispersion can be continuously tuned via excitation power, highlighting the high degree of control and adaptability of these self-hybridized polaritons. These results provide strong evidence that bare WS_2_ layers can support nonradiative EPs, opening new possibilities for integrated nanophotonic and valleytronic devices.

Cho et al. addressed the challenge of far-field detection of guided EPs by integrating a 1D PC (grating structure) into WS_2_ multilayers (Figure 10c) [172]. While guided EPs normally exist as nonradiative modes confined within the WS_2_ layer, the periodic grating structure enables momentum matching, allowing them to be coupled into the far field for optical measurement. Through angle-resolved reflectance and PL spectroscopy (Figure 10d), the authors confirm the formation of guided-mode resonances in WS_2_ gratings as thin as 10 nm. They also demonstrate that strong excitonic resonances in WS_2_ naturally lead to guided EP formation, and the grating facilitates efficient coupling of these modes into free space. This work bridges the gap between nonradiative polariton physics and practical photonic applications, paving the way for scalable, on-chip exciton-polaritonic devices using WS_2_-based nanostructures.

Zhang et al. demonstrated a WS_2_ PC as a self-resonant polariton system, eliminating the need for external cavities (Figure 10e) [173]. The 12 nm-thick WS_2_ PC supports intrinsic optical resonances, leading to anticrossing at 1.97 eV and a Rabi splitting of ∼100 meV (Figure 10f). This design achieves deep subwavelength light confinement, offering a new approach for ultra-thin polaritonic and quantum photonic devices.

These three works collectively demonstrate the feasibility and advantages of cavity-free EPs, establishing new methods to generate, manipulate, and observe polaritonic states, offering new possibilities for scalable nanophotonic devices, integrated valleytronic systems, and ultra-compact quantum photonic technologies.

**Figure 11:**
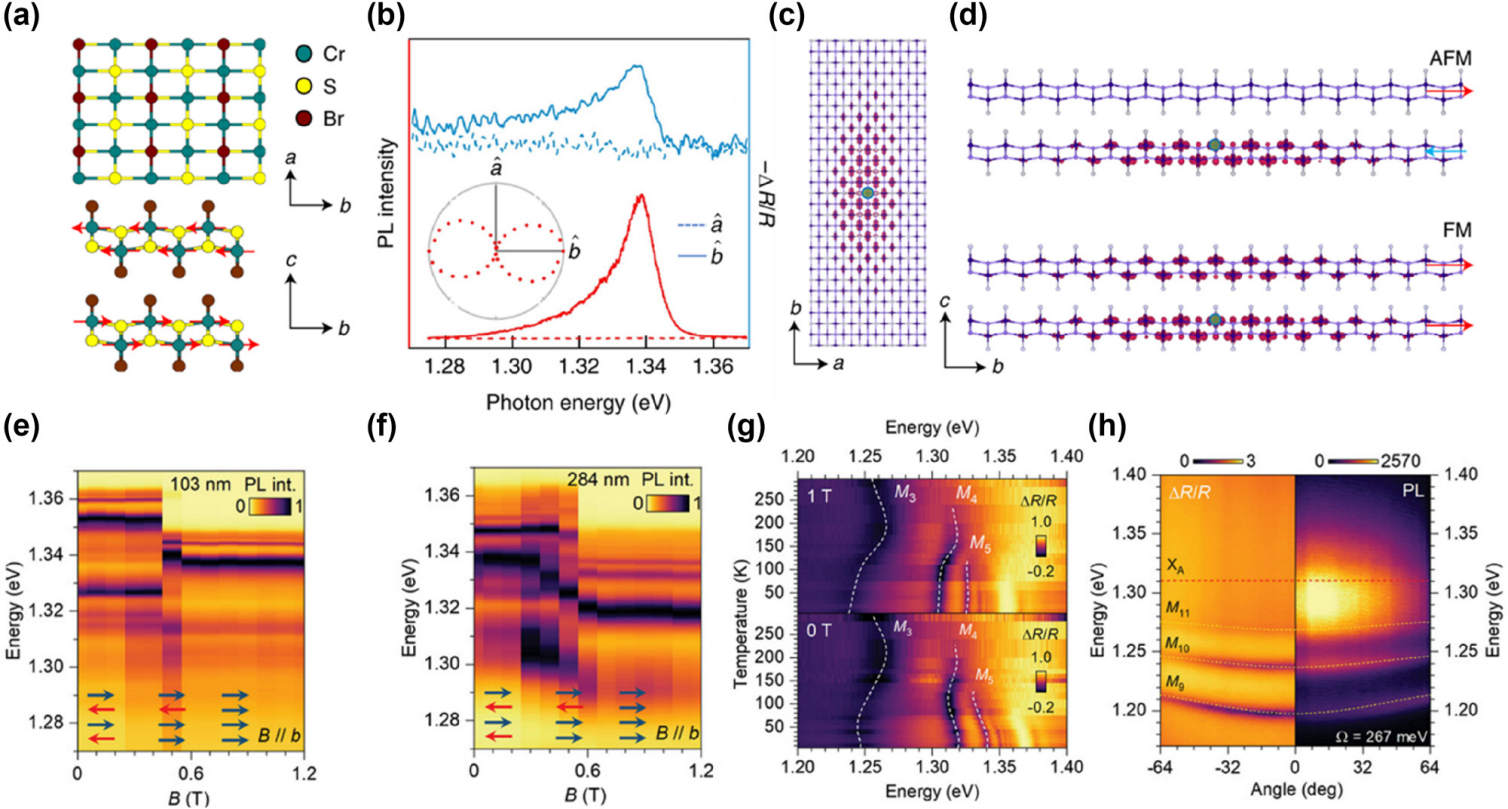
The properties and special EP characteristics of a novel 2D magnetic vdW material, chromium sulfide bromide (CrSBr). (a) The crystal and magnetic structures of CrSBr. The top image is a top view of a single CrSBr layer, depicting the 2D layered structure of the material, while the bottom image is a side view of a bilayer CrSBr, where red arrows represent the interlayer antiferromagnetic (AFM) order. (b) The differential reflectance spectra (blue) and PL spectra (red) of bilayer CrSBr with light polarized along the *b*-axis (solid lines) and *a*-axis (dashed lines). (c) Top view of the real-space wavefunction of the lowest-energy exciton in CrSBr bilayer. (d) The side view of the exciton wavefunction in the AFM bilayer (top) and ferromagnetic (FM) bilayer (bottom) state. Panel (a–d): reproduced from ref. [179]. Copyright 2021, Springer Nature. (e, f) The normalized 2D colored PL spectra of the 103 nm-thick (e) and 284 nm-thick (f) CrSBr crystals at 10 K under a magnetic field (*B*) ranging from 0 to 1.2 T. (g) The 2D reflectance spectra of the 354 nm-thick CrSBr crystal under 0 T (bottom panel) and 1 T (top panel) magnetic fields. (h) Angle-resolved reflectance and angle-resolved PL map at 298 K of the 1,260 nm-thick CrSBr crystal. Panel (e–h): reproduced from ref. [31]. Copyright 2024, Wiley-VCH.

In addition to the research on polaritons in the aforementioned TMD materials, CrSBr, as a novel 2D layered magnetic semiconductor, has attracted widespread attention recently due to its unique structural, electronic, magnetic, and optical properties. The crystal structure of CrSBr comprises chromium sulfide double layers flanked on both sides by anionic bromide layers, all separated by vdW gaps (top image of Figure 11a) [175]. This configuration allows for straightforward mechanical exfoliation down to monolayer or few-layer thicknesses. Each chromium ion resides in a distorted octahedral coordination environment formed by four sulfur atoms and two bromine atoms, which contributes to the material’s electronic and magnetic anisotropy. CrSBr exhibits A-type antiferromagnetic order below its Neél temperature of 132 K. In this magnetic state, spins align ferromagnetically within each vdW layer and antiferromagnetically between adjacent layers (bottom image of Figure 11a) [176], [177]. This material shows strong triaxial magnetocrystalline anisotropy, with the *b*-axis serving as the easy magnetic axis, the *a*-axis as the intermediate magnetic axis, and the *c*-axis being the hard magnetic axis. When a magnetic field is applied along the *b*-axis, a sharp transition to magnetic saturation occurs, which is consistent with a spin-flip transition due to large magnetocrystalline anisotropy energy [176], [177], [178]. In addition to magnetic anisotropy, CrSBr exhibits significant optical anisotropy, including PL that exhibits strong polarization dependence. The PL intensity is strongest when the incident light is polarized along the *b*-axis and weakest when polarized along the *a*-axis (Figure 11b). This indicates that the excitonic wavefunction is more delocalized along the *b*-axis than the *a*-axis (Figure 11c), in agreement with the anisotropic band structure [179]. In the AFM state, interlayer hybridization is suppressed due to antialigned spins, whereas in the FM state, electronic wavefunctions can couple between layers (Figure 11d), resulting in band splitting and a reduction in the bandgap. These computational results align well with the experimentally observed exciton redshift, highlighting the crucial role of magnetic ordering in interlayer electronic coupling [179]. The 2D nature and high stability of CrSBr make it an ideal platform for studying low-dimensional quantum physics and correlated electronic phenomena, including research into magnetic correlations at low temperatures [180], EPs [31], [181], [182], and other complex physical phenomena.

CrSBr flakes with sufficient thickness form microcavities, enabling strong exciton–photon interactions along the *b*-axis, leading to self-hybridized EPs [181], [182]. Li et al. demonstrated stable EP behavior in CrSBr crystals, investigating the effects of thickness, magnetic field, and temperature [31]. Figures 10f and 11e show PL spectra of CrSBr crystals (108 nm and 203 nm) at 10 K under magnetic fields. At 0.5 T, a redshift occurs, indicating a transition from AFM to FM. Figure 11g shows a redshift in EP energy at 1 T, which decreases with increasing temperature. Figure 11h shows the angle-resolved reflectance and angle-resolved PL imaging for a much thicker CrSBr crystal (1,260 nm) at room temperature (298 K), indicating a Rabi splitting energy of 267 meV, confirming ultrastrong coupling. CrSBr’s magnetic tunability and strong coupling at room temperature make it ideal for photonic devices like tunable filters, photodetectors, and light sources, with promising near-infrared applications.

## 4 SPE from 2D vdW materials

In recent years, quantum photonics has brought transformative advancements across multiple fields, including secure communication [183], computation [184], [185], and sensing [186]. Single-photon sources play a vital role in this field by enabling precise control over light–matter interactions at the nanoscale. For 2D SPE, three key aspects (emission origin, defect engineering, and rational design) play crucial roles in optimizing the efficiency and stability of quantum light sources.

*Emission origin*—In the simplest case, an SPE can be modeled as a two-level system, defined by its transition energy and dipole matrix element, as mentioned above. SPE in 2D materials originates from quantum-confined excitons that become trapped in localized defect states or strain-induced potential wells. The fundamental requirement for SPE is that only one exciton can occupy the localized state at a time, preventing multiphoton emission. Defect-state emission occurs when atomic-scale vacancies or substitutional atoms introduce deep mid-gap states, trapping excitons at these defect sites where they undergo radiative recombination, emitting single photons. A notable example is nitrogen vacancies in hBN, which enable room-temperature SPE due to their deeply localized states [17]. Strain-induced exciton localization, on the other hand, modifies the local bandgap, forming potential wells that capture excitons. These localized excitons recombine to produce narrow and stable SPE peaks. A prominent example is strain-induced quantum dots (QDs) in WSe_2_, which serve as efficient single-photon sources. Lastly, in twisted bilayers, such as MoSe_2_/WSe_2_ heterobilayers [140], periodic Moiré superlattices create spatially confined exciton potential minima. These potential traps localize excitons, facilitating deterministic SPE, making them highly attractive for quantum photonics applications.

*Defect engineering*—Defect engineering in 2D single-photon emitters involves the intentional creation, modification, and control of atomic-scale defects to tailor optical and electronic properties, enabling SPE. In this process, defects introduce localized electronic states within the material’s bandgap, trapping excitons and ensuring that photons are emitted one at a time, a crucial feature for quantum applications. Various methods have been developed to create and control these defects in TMDs and hBN. Ion beam irradiation, using focused helium or neon ion beams, displaces atoms from the lattice to form vacancies, which act as exciton traps in materials like MoS_2_ [187] and WSe_2_ [188]. Similarly, high-energy electron beam irradiation modifies atomic arrangements, generating stable defect sites such as nitrogen and boron vacancies in hBN, known for their robust room-temperature quantum emission [189], [190]. Chemical functionalization and annealing provide another avenue for defect control, where selective exposure to oxidizing or reducing agents modifies defect states, and thermal annealing either activates or passivates these states to enhance emission stability [191]. Additionally, strain engineering, achieved through nanopillars or wrinkled substrates, alters the local band structure, forming quantum-dot-like potential wells that localize excitons and enhance SPE. In twisted bilayers of TMDs, Moiré superlattices naturally create periodic exciton potential traps, leading to deterministic SPE [140]. These advanced defect engineering techniques enable precise control over quantum emitters, paving the way for scalable and high-purity single-photon sources essential for quantum communication, computation, and sensing applications.

*Rational design*—To engineer high-performance SPEs, several strategies have been employed. Strain and electric field tuning have been demonstrated to dynamically modulate emission wavelengths, while gate-tunable heterostructures, such as graphene/hBN [192], enable controlled charge-state switching for enhanced emission control. Coupling these emitters to optical cavities significantly boosts their brightness and extraction efficiency [193], [194], [195]. Additionally, charge-state control through electrical gating allows for on-demand switching of emission, improving stability and robustness [196]. For large-scale applications, deterministic positioning of SPEs is crucial, and plasmonic array offers a promising approach to creating ordered SPE arrays for integrated quantum photonic networks [197].

Effective single-photon sources must exhibit high brightness, purity, and indistinguishability to support quantum technologies like secure quantum communication and quantum computing. Brightness quantifies the probability of emitting a single photon upon excitation, purity refers to the source’s ability to emit only one photon per event, and indistinguishability ensures that each photon remains consistent across different degrees of freedom [198]. Each of these factors is essential to applications requiring interference, like quantum computing, where photon consistency is crucial for multiphoton interactions [199], [200]. Therefore, the selection of materials for single-photon sources requires multifaceted consideration. While traditional platforms like QDs [201], [202], [203], [204], [205], [206] and nitrogen-vacancy [207], [208], [209], [210] centers in diamonds have set benchmarks, novel 2D vdW materials such as TMDs and hBN are rapidly gaining attention.

TMDs are particularly notable for their strong electron–hole binding energy, which supports SPE through excitons trapped by localized defects or strain. Since the demonstration of SPE from TMDs in 2015 [18], [19], [211], [212], [213], numerous methods have emerged to enhance their emission efficiency. For instance, coupling TMDs to plasmonic nanostructures like GaP nano-antennas has achieved brightness levels near 0.86 and quantum efficiency of approximately 80 % [214]. These modifications allow TMDs to produce bright, high-purity SPEs with relatively fast emission rates, making them suitable for low-temperature quantum information processing applications.

Due to the wide bandgap and remarkable resistance to decoherence, hBN enables stable SPE even at room temperature. The single-photon emitters in hBN, often linked to boron or nitrogen vacancies, are robust even under ambient conditions, making them an attractive candidate for quantum photonics [215], [216], [217], [218], [219], [220], [221], [222], [223], [224].

One of the major challenges in single-photon source research is achieving tunability to ensure that the light source meets the demands of quantum applications while enhancing system stability and adaptability. In recent years, researchers have made significant advances in the tunability of single-photon sources, including strain tuning, electric field tuning, magnetic field tuning, and cavity coupling.

**Figure 12:**
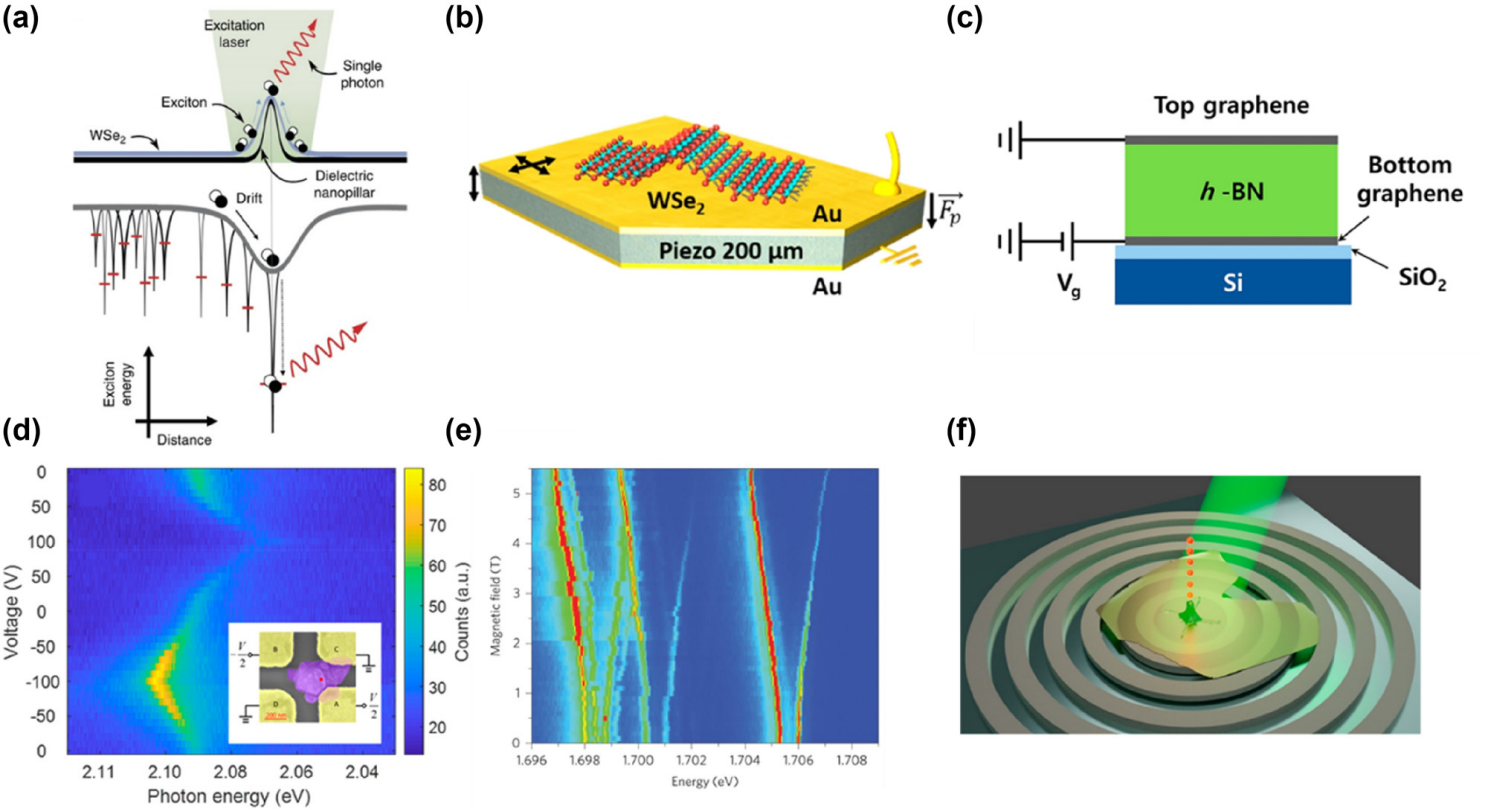
Different modulation methods of SPE from 2D vdW materials. (a) The concept of achieving strain-induced quantum emitters in atomically thin WSe_2_ using an array of nanopillars. The local biaxial strains modify the bandgap of WSe_2_ and spatially modulate the potential landscape of the 2D excitons, leading to the efficient funneling of photoexcited excitons toward the lower energy states at the strain-tuned sites, eventually forming efficient single-photon quantum emitters. Reproduced from ref. [225]. Copyright 2017, Springer Nature. (b) Schematic of a hybrid 2D-semiconductor-piezoelectric actuator device with an integrated WSe_2_ monolayer. Reproduced from ref. [226]. Copyright 2019, American Chemical Society. (c) The device schematic of multilayer hBN sandwiched between top and bottom few-layer graphene electrodes. This device is designed to tune the emission energy of single-photon emitters through the Stark effect by applying an out-of-plane electric field. Reproduced from ref. [232]. Copyright 2018, American Chemical Society. (d) The shift of the zero-phonon line PL spectrum’s central wavelength of a single-photon emitter in hBN under different applied voltages. The inset shows the setup for applying voltage between electrodes A and B. Reproduced from ref. [233]. Copyright 2019, American Chemical Society. (e) The PL intensity plot of five single quantum emitters as a function of the applied magnetic field, ranging from 0 T to 5.5 T. Reproduced from ref. [19]. Copyright 2015, Springer Nature. (f) The design and characteristics of the Purcell-enhanced single-photon source based on a circular Bragg grating cavity. Reproduced from ref. [236]. Copyright 2021, American Chemical Society.

As shown in Figure 12a, Branny et al. used nanoscale strain engineering to create local strain perturbations in monolayer and bilayer WSe_2_ [225]. They placed lithographically patterned nanopillars under atomically thin WSe_2_ flakes, which induced significant localized elastic strain at the nanopillar sites. This strain modified the band-gap of the semiconductor, funneling excitons to these smaller band-gap regions, leading to the formation of highly pure single photon-emitting quantum emitters. The use of nanopillars enabled the precise positioning of these quantum emitters. By adjusting the nanopillar dimensions, they improved both the yield of emitter formation and their positioning accuracy. This strain-based control method is well-suited for creating scalable and structured arrays of single-photon emitters.

In 2019, Iff et al. demonstrated a hybrid structure combining a 2D semiconductor and a piezoelectric device to control the emission energy of single-photon emitters in WSe_2_ monolayers (Figure 12b) [226]. Specifically, they used piezoelectric actuators to apply strain fields, allowing for the energy of localized excitons to be tuned by up to 18 meV. The strain was applied via an electric field across the sample, which consisted of a mechanically exfoliated WSe_2_ monolayer transferred onto a piezoelectric plate. Using PL spectroscopy, the researchers observed both redshifts and blueshifts in emission energy under strain modulation. This study provides a new approach for creating energy-tunable single photon sources.

The emission energies of different emitters are often inhomogeneous, which poses a significant challenge for quantum information processing [227], [228], [229]. Therefore, a method to tune the emission energy of individual emitters is required. The Stark effect refers to the phenomenon in which the energy levels of electrons within atoms or molecules shift or split under the influence of an external electric field [230], which is an effective approach that enables the tuning of SPE energies through the application of an external electric field [231]. Noh et al. combined exfoliated hBN flakes with graphene to create heterostructures with top and bottom graphene electrodes (Figure 12c) [232]. Applying a voltage generated a vertical electric field to control defect centers in hBN, resulting in Stark-induced tuning of the emission with a maximum shift of 5.4 nm per GV/m. This work showed electric field control of hBN single-photon emitters. Xia et al. later designed a four-electrode nanodevice for more flexible electric field control, achieving a large Stark shift of 43 meV/(V/nm), the highest reported at room temperature [233]. Figure 12d shows the zero-phonon line PL intensity as a function of the applied voltage, with a Stark shift up to 31 meV, demonstrating reversible tuning of the single-photon emitter energy by the electric field.

Meanwhile, the emission characteristics of single-photon sources can also be controlled by magnetic fields. He et al. introduced defect-localized excitons in WSe_2_ monolayers as single quantum emitters with narrow optical emission linewidths (∼130 μeV), much narrower than delocalized valley excitons [19], [234]. These emitters exhibit two nondegenerate, linearly polarized transitions at zero magnetic field [235]. Applying a magnetic field causes the polarization to transition from linear to circular as it competes with electron–hole exchange interactions, leading to the Zeeman effect (Figure 12e). The magnetic field splits the energy levels of each emitter and controls the polarization state of emitted photons, enhancing the flexibility of single-photon sources for quantum information applications like quantum key distribution.

To further improve the emission efficiency of single-photon sources, the application of optical cavities is an essential topic. As shown in Figure 12f, Iff et al. proposed a deterministic single-photon source based on the integration of WSe_2_ QDs with a circular Bragg grating cavity [236]. The cavity enhances SE through the Purcell effect, thereby improving the efficiency and scalability of the single-photon source. The use of the circular Bragg grating cavity enables deterministic placement of QDs and strain-induced formation, significantly enhancing emission. This provides a new approach for developing a single-photon emitter that is simple to manufacture, cost-effective, and performs well.

Despite the remarkable performance of 2D semiconductor single-photon sources in terms of efficient SPE and tunability, challenges remain in integrating these devices into practical quantum information processing systems. Currently, individual or small numbers of 2D single-photon sources have been validated in laboratory settings [19], [225], [226], [232], [233], [236], but these isolated devices struggle to meet the demands for high integration and stability in real-world applications. To solve this problem, the measures of combining 2D semiconductor single-photon sources with mature chip manufacturing technologies are beginning to be explored. Through on-chip integration, device miniaturization and mass production can be realized, enhancing system reliability and scalability. This advancement paves the way for the practical application of quantum information technologies and represents a key direction for future research.

**Figure 13:**
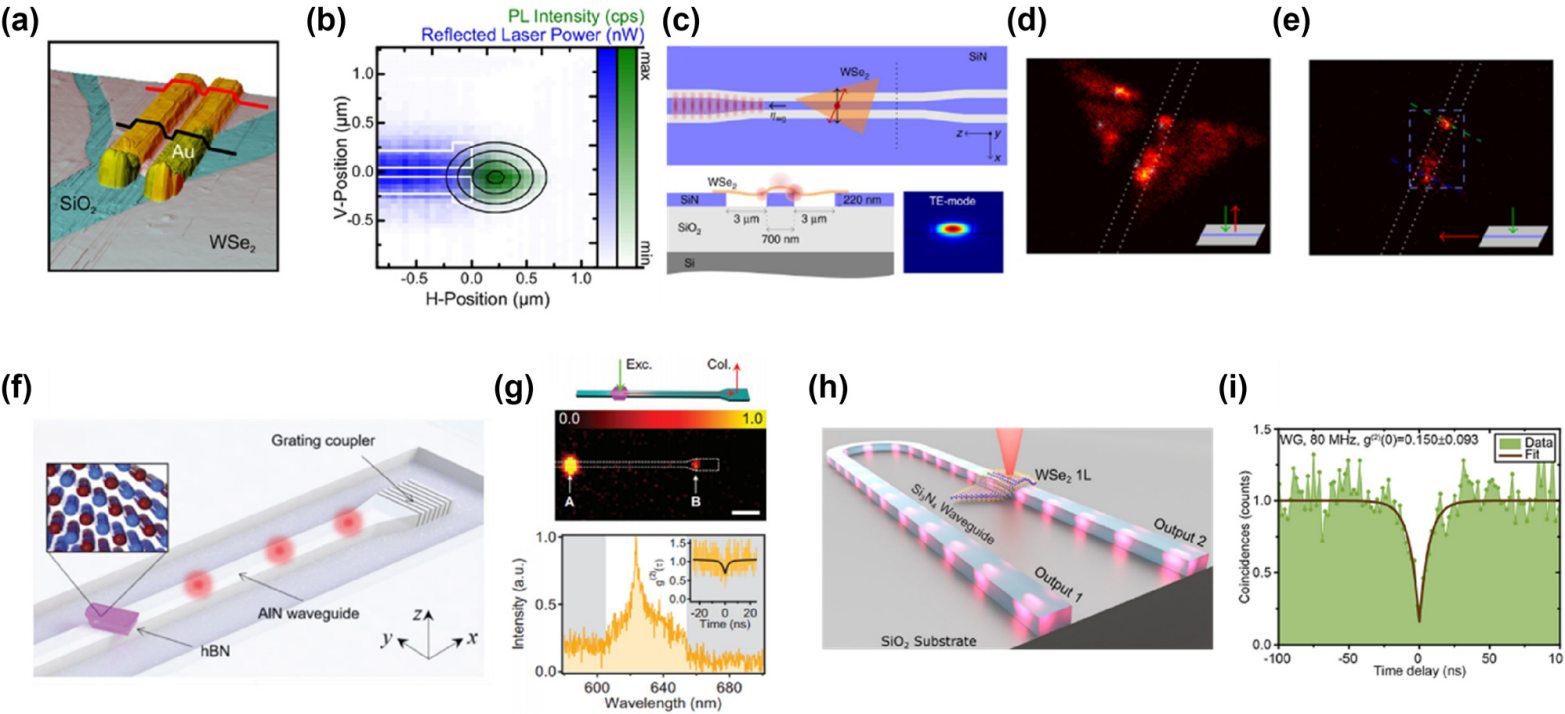
On-chip integration of 2D SPE emitters. (a) False-color perspective view of an atomic force microscope image of the combined plasmonic waveguide and monolayer WSe_2_ system. (b) The relative position of the QD and waveguide as obtained from high-resolution PL and laser reflectivity scans. Panel (a–b): reproduced from ref. [237]. Copyright 2018, American Chemical Society. (c) Upper panel: schematic of the device integrating a WSe_2_ flake onto a SiN waveguide. Lower panel: a cross-sectional view of the sample (left) and a cross-sectional mode profile (at 750 nm) of the waveguide (right). (d) PL scan from the top of the sample. (e) Waveguide-coupled PL scan collected through the fiber. Panel (c–e): reproduced from ref. [242]. Copyright 2019, Springer Nature. (f) Schematic of the hybrid system, where an hBN flake is integrated onto an AlN waveguide. (g) Top panel: schematic of the nonlocal collection scheme. Emission from the grating coupler of the waveguide (spot B) could be collected. Bottom panel: PL spectrum collected from spot B (the grating coupler). The inset in the figure shows the second-order autocorrelation function *g* ^(2)^(*τ*), panel (f–g): reproduced from ref. [248]. Copyright 2019, Wiley-VCH. (h) Schematic of the coupled WSe_2_ monolayer on the Si_3_N_4_ waveguide. (i) The result of the second-order autocorrelation measurement conducted through waveguide output 1 (*g* ^(2)^(0) = 0.150 ± 0.093). Panel (h–i): reproduced from ref. [249]. Copyright 2021, American Chemical Society.

Blauth et al. integrated WSe_2_ monolayer quantum emitters with metal plasmonic waveguides for nanoscale single-photon generation and routing (Figure 13a) [237]. The emitter was positioned close to the waveguide edge (Figure 13b), enabling on-chip single-photon sources suitable for quantum information and optical communication. Strain engineering and hBN encapsulation could enhance coupling efficiency and emitter quality [238], [239], [240], [241]. And in 2019, Peyskens et al. used a dry-transfer method to integrate WSe_2_ flakes onto SiN waveguides, achieving efficient light confinement for on-chip transmission (Figure 13c) [242]. Confocal PL scans (Figure 13d) and PL collected via fiber (Figure 13e) confirmed enhanced emission on the waveguide, highlighting its scalability for quantum photonic chips without complex postprocessing.

As previously mentioned, when discussing room-temperature single-photon sources, it is imperative to consider hBN, as it is capable of producing narrowband SPE across a wide temperature range, including room temperature [17], [243], [244], [245], [246], [247], thereby making it an ideal material for quantum emitters. hBN emitters integrated with AlN waveguides demonstrated room-temperature SPE and coupling (Figure 13f and g) [248]. Photons successfully coupled into the waveguide and were transmitted through the grating coupler, confirming SPE.

In 2021, Herranz et al. utilized SiN waveguide edges to create strain-induced single-photon emitters in WSe_2_, achieving effective waveguide coupling (Figure 13h) [249]. Emission analysis showed *g* ^(2)^(0) = 0.150, confirming SPE through waveguide coupling (Figure 13i). These results confirm the single-photon nature of the emission, indicating that, despite background noise, the quality of SPE is maintained through waveguide coupling.

## 5 Other types of 2D photonic source

In the preceding sections, we have comprehensively reviewed lasers and single-photon sources based on 2D vdW materials, highlighting their exceptional performance in optics and quantum optics, such as high gain, tunability, and stability at room temperature. These attributes establish 2D vdW materials as strong candidates for next-generation optoelectronic devices. However, the optical properties of 2D vdW materials extend beyond the domain of linear optics; they also exhibit significant potential in nonlinear optics. Notably, phenomena such as HHG and P-band emission have demonstrated unique advantages in 2D vdW materials.

The HHG response in 2D vdW materials makes it suitable for integrated nonlinear nanophotonic devices, such as optical modulators [250] and optical switches [251]. HHG can produce significant nonlinear optical responses under low-intensity laser conditions [252], which helps in the realization of more efficient and compact optical devices. In recent years, HHG in 2D vdW materials has attracted significant research interest [253], [254], [255]. However, enhancing HHG intensity and achieving more effective control over the process remain key challenges that warrant further exploration.

**Figure 14:**
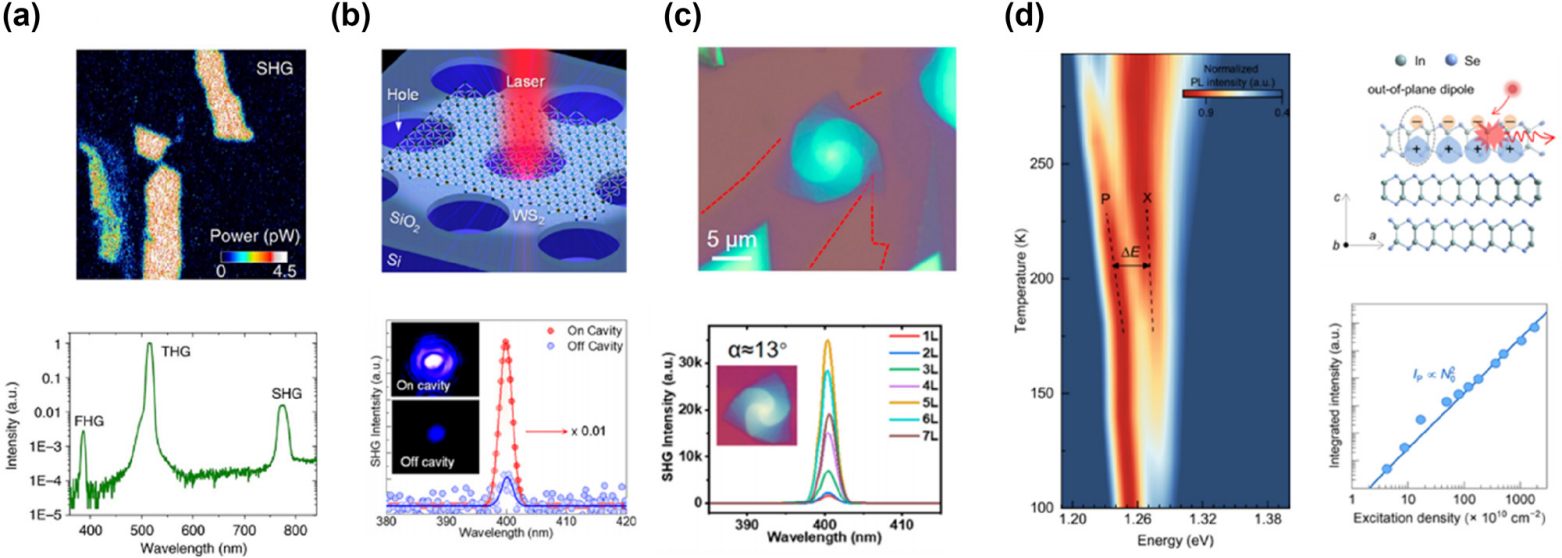
Tuning of high-order harmonic generation and exciton–exciton scattering induced P-band emission. (a) Top panel: the SHG map of MoS_2_ flakes. Bottom panel: the PL spectrum of the nonlinear signal from the monolayer MoS_2_. Reproduced from ref. [256]. Copyright 2017, Springer Nature. (b) Top panel: schematic of the WS_2_ monolayer placed on a silicon substrate with holes. Bottom panel: the SHG spectra collected from the WS_2_ monolayer on cavity (red) and off cavity (blue) under excitation with an 800 nm fs pulsed laser. Insets: the microscope image of SHG emission from the WS_2_ monolayer in the on-cavity and off-cavity regions. Reproduced from ref. [266]. Copyright 2022, American Chemical Society. (c) Top panel: the optical microscopy images of a right-handed supertwisted WS_2_ spiral with a twist angle of approximately 17°. Bottom panel: the SHG spectra of supertwisted WS_2_ spirals with a twist angle of around 13°, demonstrating the SHG response for different layer numbers. The SHG intensity gradually increases from 1 to 5 layers and then drops rapidly from 6 to 7 layers, indicating different nonlinear optical properties in the twisted structure depending on the layer number. Reproduced from ref. [267]. Copyright 2024, American Chemical Society. (d) Left panel: the PL spectra of an exfoliated InSe crystal under different temperatures, ranging from 298 K to 100 K. Right panel (upper): the side view of the γ-phase InSe crystal structure, highlighting the out-of-plane dipole moment and the inhomogeneous charge distribution of excitons. Right panel (lower): the relationship between the P-band emission intensity and the excitation density. Reproduced from ref. [27]. Copyright 2023, American Chemical Society.

Säynätjoki et al. investigated the nonlinear optical responses of monolayer MoS_2_, focusing on third-harmonic generation (THG) and fourth-harmonic generation [256]. The second-harmonic generation (SHG) map (Figure 14a, top panel) shows SHG in monolayer MoS_2_ but not in bilayer MoS_2_, as SHG requires noncentrosymmetry, which is absent in bilayers [25], [257]. One of the notable discoveries in this study is the observation that THG is significantly stronger than SHG (Figure 14a, bottom panel), contrary to conventional expectations where the efficiency of higher-order harmonic processes is typically weaker [258], [259]. The key reason behind this unexpected result lies in the trigonal warping of the electronic band structure of MoS_2_. The lack of spatial inversion symmetry plays a critical role – though it alone is insufficient for generating SHG, the combination of noncentrosymmetric band structure and trigonal warping facilitates efficient harmonic generation [93], [260], [261], [262], [263], [264], [265].

In terms of integration with microcavities, Shi et al. enhanced SHG in WS_2_ by placing a monolayer over a patterned silicon substrate to create a Fabry–Pérot microcavity, amplifying SHG by up to 1,580 times (Figure 14b) [266]. Coupling WS_2_ with cavity modes at the excitation wavelength (820 nm) resulted in electric field amplification and improved directionality (with a divergence angle of ∼5°), crucial for applications in integrated photonics and optoelectronics.

Recently, Tong et al. demonstrated the synthesis of supertwisted WS_2_ spirals and their effect on nonlinear optical properties [267]. Using a water-assisted CVD method, the spirals were grown on non-Euclidean surfaces to achieve different twist angles (Figure 14c, top panel). The study observed an oscillatory dependence of SHG intensity on the layer number of the supertwisted spirals, which is attributed to phase matching of nonlinear dipoles within different layers (Figure 14c, bottom panel). Additionally, varying the twist angle resulted in different periodic structures that enabled inversion symmetry breaking, leading to an enhancement in the SHG signal by a factor of 2–136 compared with a single-layer structure.

As an important aspect of nonlinear optics in 2D vdW materials, p-band emission has received much research attention in recent years, especially concerning its related properties at room temperature. P-band emission is a superlinear and low-coherence light emission phenomenon driven by exciton–exciton scattering [268], [269], providing a low-coherence, high-intensity light source at low power, holding promise for applications in speckle-free imaging [270], [271], frequency-resolved lidar [272], and interferometric sensing [273], [274].

Liang et al. investigated exciton dynamics and P-band emissions in exfoliated InSe, revealing P-band emission under CW excitation at low excitation density (∼10^10^ cm^−2^), unlike typical superlinear emissions requiring high densities and pulsed modes [275], [276], [277], [278], [279]. This is enabled by strong exciton–exciton scattering, due to enhanced spatial confinement and unique material properties of InSe. The γ-phase InSe structure with an out-of-plane dipole orientation supports efficient exciton scattering (right panel of Figure 14d, upper) [29], [143], [144], [147]. P-band intensity followed a quadratic dependence on exciton density (right panel of Figure 14d, lower), confirming pair scattering as the emission source. Cooling caused a blue shift in P-band and X-peak energies, indicating increased exciton stability and reduced phonon scattering. Meanwhile, energy difference between P-band and X-peak energies increases from 29.1 meV to 49.8 meV (Figure 14d, left panel), which is attributed to increased kinetic energy loss during scattering as the temperature rises. Higher temperatures result in increased kinetic energy for the excitons, which means that more energy is converted to other forms, such as heat or phonon energy, during exciton–exciton scattering, leading to an increased energy difference, which supports the exciton–exciton scattering model [269], [280]. This work demonstrates strong excitonic interactions in InSe, paving the way for efficient low-coherence light sources and near-infrared optoelectronic devices.

## 6 Electrically driven 2D photonic sources

The implementation of electrical pumping operation is a significant milestone toward the practical application of emergent 2D vdW light sources [281]. During the device structure design, the key step is to enable effective injections of charge carriers into 2D vdW semiconductors to promote light emission, as has always been one of the core issues concerned in the traditional semiconductor field [282], [283], [284]. So far, the widely used carrier injection techniques mainly include electrostatic doping, tunneling junction, band alignment engineering, and alternating current driven injection [285]. In the early stages of research, several groups developed various types of LEDs based on 2D vdW semiconductors, and the external quantum efficiency (EQE) of such exciton emission dominated devices at room temperature reached up to 5 % [197], [286], [287], [288], [289], [290], [291].

A significant issue is that the external quantum efficiency (EQE) of early 2D LEDs (up to ∼5 %) is significantly lower than the PL quantum yield (approximately 20 %) of the intrinsic 2D materials. The potential causes may include the exacerbation of nonradiative recombination channels, such as scattering caused by defects at the device interfaces, exciton–exciton annihilation at high carrier concentrations, nonradiative recombination involving exciton complexes at high doping levels, ineffective or unbalanced carrier injection, carrier leakage, and low optical outcoupling efficiency [290], [292], [293], [294], [295]. While addressing these issues simultaneously to further enhance the EQE remains challenging, some research groups have made attempts. For example, in 2020, Kwon et al. demonstrated a WSe_2_-based light-emitting transistor, which included a monolayer WSe_2_ channel and graphene contacts, coupled with two separate top metal gates [296]. By adjusting the contact barrier height, the type and density of injected charge carriers could be independently controlled to achieve balanced injection, resulting in bright emission near 750 nm with a high peak EQE of ∼6 % at room temperature. Similarly, in 2024, Shin et al. also utilized a double-gate structure (containing graphene and silicon gates) to develop a WSe_2_-based light-emitting transistor with balanced electron and hole injection [297]. Furthermore, with the help of a local graphene gate, electrons and holes could flow into the 1D region to form neutral excitons. The in-plane electric field within the 1D region effectively confines the neutral excitons and expels charged excitons through charge interaction, thus enhancing the efficiency of radiative recombination dominated by neutral excitons. The demonstrated device exhibits an improved maximum EQE of ∼8.2 % at room temperature. To achieve high-performance mid-infrared LEDs, Gupta et al. placed the light-emitting BP/MoS_2_ heterostructure on Al_2_O_3_/Au to form a vertical resonant cavity, which simultaneously enhances the SE rate via Purcell effect and light outcoupling efficiency by appropriately designing the cavity length [298]. In addition, a transparent indium tin oxide conductive layer at the top can reduce the parasitic resistance while having almost no impact on light output. The measured operating wavelength is 3.65 μm with an EQE of 4.43 %, and the overall performance exceeds that of commercial mid-infrared LEDs, comparable to the interband cascade lasers.

**Figure 15:**
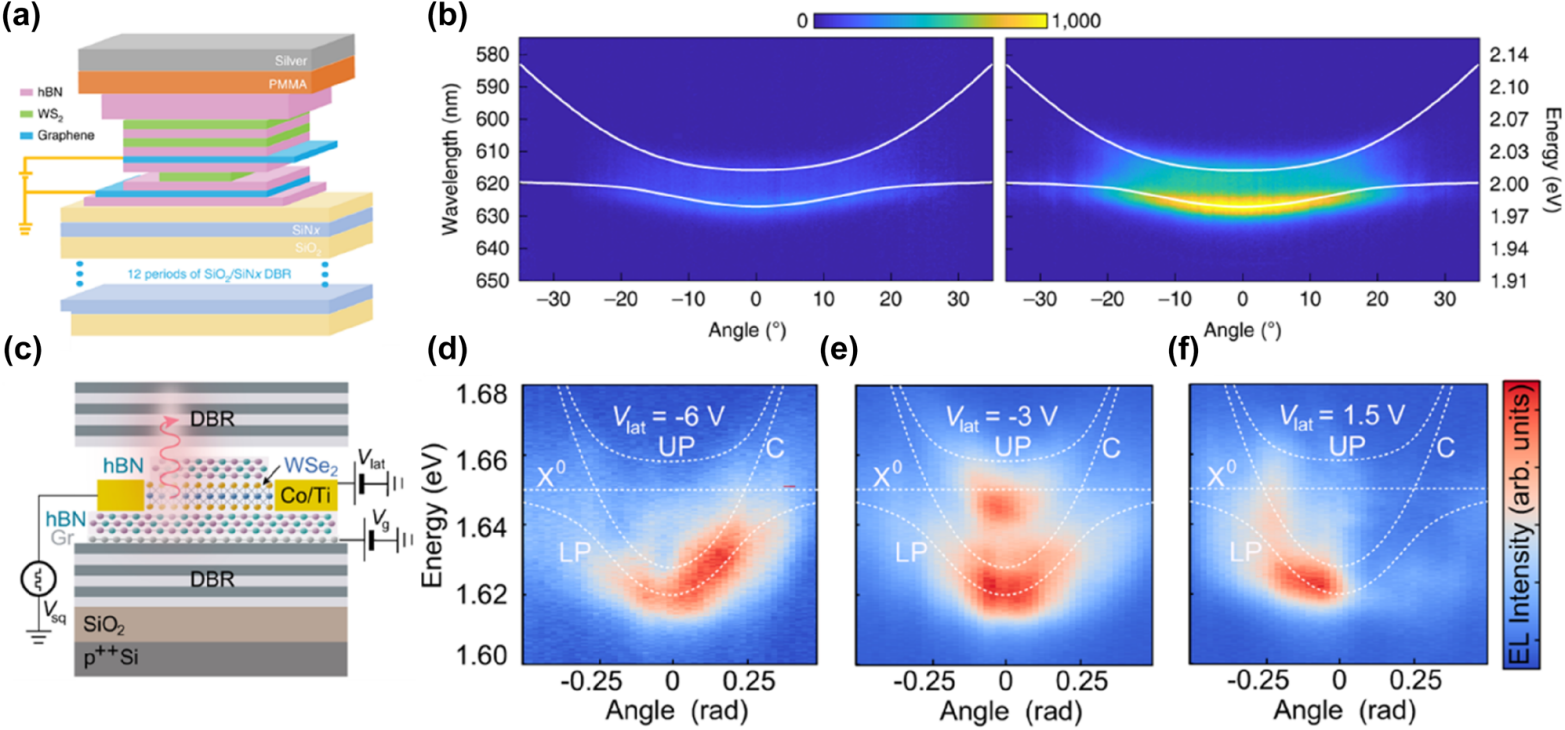
Electrically pumped EP LEDs. (a) Schematic of an electrically pumped EP LED with different layers of the vdW heterostructure embedded inside a bottom DBR and top silver mirrors. (b) Angle-resolved PL (left panel) and electroluminescence (right panel) of the device in (a). Reproduced from ref. [299]. Copyright 2019, Springer Nature. (c) Schematic of an electrically pumped EP LED with electrical control of polarization and emission angle. (d–f) Angle-resolved electroluminescence spectra at the lateral voltage of −6 V (d), −3 V (e), and 1.5 V (f) of the device in (c). Reproduced from ref. [301]. Copyright 2022, Springer Nature.

Compared to weakly coupled devices, EP LEDs in the strong coupling regime offer higher regulatory flexibility. Gu et al. reported the first room-temperature EP LED based on 2D vdW materials, where multiple WS_2_ monolayers serve as the active layers to enhance exciton density, hBN serves as the tunneling spacer layer, and graphene serves as the transparent conductive layer (Figure 15a [299]). The above structure is embedded in the bottom DBR (consisting of 12 pairs of SiO_2_/SiN, with a metal electrode deposited on the surface) and the top silver/PMMA film to detect the EP emission. Angle-resolved electroluminescence spectra indicate the emission follows the dispersion feature of EPs, with the emission angle confined within ±15° (Figure 15b). The device displays an EQE of 0.1 %, comparable to the performance of organic molecule- and carbon nanotube-based EP LEDs reported at the time.

Since EPs possess both excitonic and photonic characteristics, real-time tuning of the emission properties can be achieved by electrically controlling the carrier properties [300]. Marin et al. further demonstrated an EP LED with room-temperature electrical control of emission polarization (polarization ratio ranging from 20 % to −20 %) and emission angle (from negative to positive [301]). The working principle involves applying a voltage to the electrode deposited above the WSe_2_ monolayer, which alters the band alignment and the overall external electric field, thereby modulating the in-plane carrier velocity and spatial distribution (Figure 15c). Through strong exciton–photon coupling, the change in the in-plane exciton momentum is inherited by the EP and converted into different photon emission angles (Figure 15d–f). Simultaneously, the change in the exciton momentum also alters the exciton energy at the *K* and *K*′ points, leading to a change in the valley EP population and strong valley polarization.

**Figure 16:**
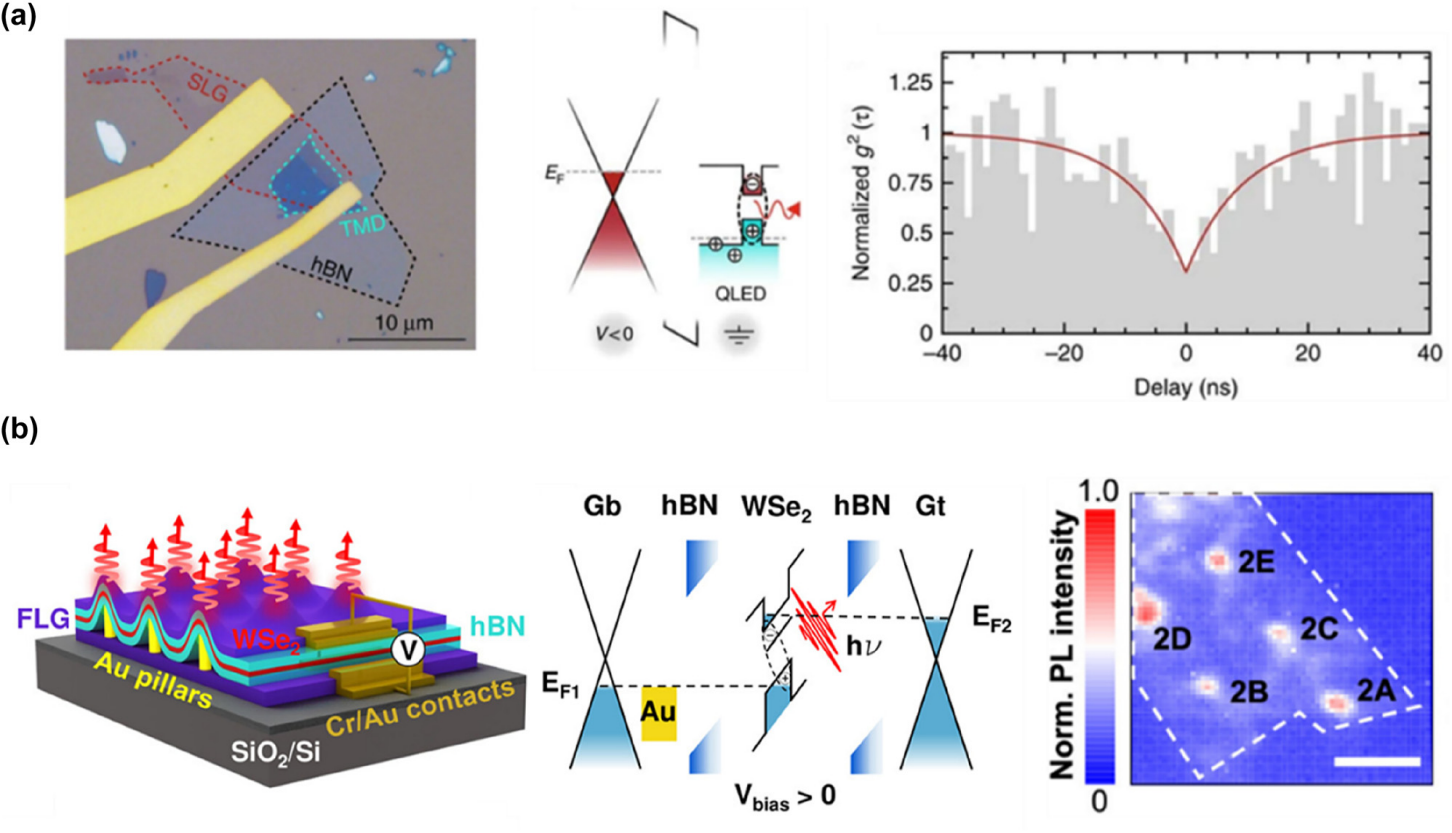
Electrically pumped single-photon sources. (a) Left panel: optical image of an electrically pumped single-photon source including Au/WSe_2_/hBN/graphene/Au on the SiO_2_/Si substrate. Middle panel: energy band diagram with applied bias. Right panel: intensity-correlation function of the electroluminescence signal. Reproduced from ref. [303]. Copyright 2016, Springer Nature. (b) Left panel: schematic of an electrically pumped single-photon source, including graphene/hBN/WSe_2_/hBN/Au pillars/graphene. Middle panel: energy band diagram with applied bias. Right panel: electroluminescence mapping over the active region. Reproduced from ref. [308]. Copyright 2023, American Chemical Society.

Furthermore, achieving electrically pumped single-photon sources is critical for the development of practical on-chip quantum emitters [302]. An early example was reported by Berraquero et al. where the device is based on a single tunneling junction including Au/WSe_2_/hBN/graphene/Au on a SiO_2_/Si substrate (Figure 16a [303]). Electroluminescence spectra at 10 K reveal several narrow emission peaks with linewidths ranging from 0.8 to 3 nm in the 750–850 nm range. The intensity-correlation function exhibits the antibunched nature with a *g* ^(2)^(0) of 0.29 ± 0.08, expected for a single-photon source. Using WS_2_ with a larger bandgap, the SPE wavelength can be extended from the near-infrared to the visible range (∼640 nm). At the same time, other research groups also observed similar electrically pumped single defect emission using tunneling junctions [304], [305].

For untreated materials, defects are usually randomly distributed, so another key issue is to customize the position of the single-photon emitter. Advanced strategies include using atomic force microscopy or scanning tunneling microscopy tips to generate on-demand atomic-level defects to localize excitons [306], [307]. Recently, Guo et al. introduced an array of Au nanopillars embedded in the WSe_2_ based device to simultaneously inject carriers and generate ordered strain (Figure 16b), ultimately producing a site-controlled electrically injected SPE with a *g* ^(2)^(0) of 0.32 ± 0.01 [308].

It is worth noting that electrically driven SPE has only been realized in TMD materials, which primarily results from excitons bound to defects with small binding energies and only operates at cryogenic temperatures. Besides, the observed SPE is generally accompanied by strong emission backgrounds associated with other excitonic processes, leading to a relatively low single-photon purity. The hBN has acted as a potential candidate for room-temperature high-purity SPE, but achieving effective electrical injection remains an unresolved challenge [229], [309].

## 7 Conclusion and outlook

In this review, we provide an in-depth introduction of light-emission properties and advancements in 2D vdW materials, specifically focusing on various light sources, such as lasers, single-photon sources, and nonlinear optical applications. The review highlights several aspects of 2D vdW materials, including their unique excitonic properties, the principles of light emission, and the development of semiconductor lasers based on these materials. It covers advancements in intralayer and interlayer exciton lasers, cavity-free laser systems, and EP emissions, emphasizing the integration potential of SPE sources in on-chip systems and exploring the nonlinear optical properties, like HHG and P-band emission. Lastly, we introduce electrically pumped light sources.

Moving forward, in the field of lasers, optimizing the laser structure of 2D vdW materials offers the potential to significantly lower the lasing threshold, enabling the development of low-power, continuous-wave laser sources. These sources are particularly suited for portable devices and energy-critical applications, such as biosensors and on-chip communication. The EP interactions inherent in 2D vdW materials are ideally suited for on-chip photonic integration, which can greatly enhance the efficiency and miniaturization of photonic systems, with wide-ranging applications in optical communication and quantum information processing. In the realm of single-photon sources, 2D vdW materials hold great promise for high-purity single-photon generation, supporting advances in quantum communication and quantum computing. As material stacking technologies continue to progress, the role of 2D vdW materials in the field of quantum information will expand further. Additionally, the nonlinear optical properties of 2D vdW materials provide new opportunities for creating compact, tunable photonic devices. Furthermore, the unique electronic and optical characteristics of 2D vdW materials allow for integration into multifunctional, complex devices, enabling the combination of various functions such as light emission, detection, and modulation. This integration could significantly reduce system costs while enhancing device performance. At the same time, 2D vdW materials exhibit excellent stability at room temperature, allowing them to operate across a wider range of temperatures, making them ideal for diverse applications, from space missions to industrial environments, without the need for additional cooling.

However, realizing these prospects presents several technical challenges. Current fabrication techniques for 2D vdW materials, such as mechanical exfoliation, are limited in terms of scalability and cannot meet commercial production demands. To enable the widespread use of 2D vdW materials, reliable large-scale production methods, such as CVD, must be developed. These methods need to ensure the production of high-quality, defect-free materials, while also allowing for precise control over layer thickness and composition. Additionally, the sensitivity of 2D vdW materials to external factors such as electric and magnetic fields make it difficult to achieve precise and stable control over their properties. Maintaining the stability of these properties in varying environmental conditions is a key challenge for improving the reliability of devices. 2D vdW materials are often integrated with other materials or substrates, but the interface interactions between them can degrade performance, leading to a loss of optical and electronic properties. Therefore, optimizing these interfacial interactions and employing techniques such as surface passivation or encapsulation are essential for enhancing device performance. Despite the significant potential of 2D vdW materials for SPE, maintaining their stability and photon purity – especially in complex environments – remains a challenge. Controlling noise and ensuring photon purity are critical for improving their reliability in quantum information applications. Finally, many 2D vdW materials exhibit an indirect bandgap in their multilayer or bulk forms, which limit their light emission efficiency. Research focused on achieving efficient lasing or SPE in multilayer structures, or on maintaining direct bandgap characteristics in thicker layers, will be key to advancing the application of 2D vdW materials in integrated photonics.

In summary, while the potential of 2D vdW materials in photonics is immense, overcoming the current challenges in fabrication, material integration, and performance stability will be crucial for realizing their full potential in next-generation optical and quantum devices.
